# A worldwide map of swine short tandem repeats and their associations with evolutionary and environmental adaptations

**DOI:** 10.1186/s12711-021-00631-4

**Published:** 2021-04-23

**Authors:** Zhongzi Wu, Huanfa Gong, Mingpeng Zhang, Xinkai Tong, Huashui Ai, Shijun Xiao, Miguel Perez-Enciso, Bin Yang, Lusheng Huang

**Affiliations:** 1 State Key Laboratory for Pig Genetic Improvement and Production Technology, Jiangxi Agricultural University, Nanchang, China; 2 Centre for Research in Agricultural Genomics (CRAG), CSIC-IRTA-UAB-UB, Campus UAB, Barcelona, Spain; 3 ICREA, Passeig de Lluís Companys 23, Barcelona, Spain

## Abstract

**Background:**

Short tandem repeats (STRs) are genetic markers with a greater mutation rate than single nucleotide polymorphisms (SNPs) and are widely used in genetic studies and forensics. However, most studies in pigs have focused only on SNPs or on a limited number of STRs.

**Results:**

This study screened 394 deep-sequenced genomes from 22 domesticated pig breeds/populations worldwide, wild boars from both Europe and Asia, and numerous outgroup Suidaes, and identified a set of 878,967 polymorphic STRs (pSTRs), which represents the largest repository of pSTRs in pigs to date. We found multiple lines of evidence that pSTRs in coding regions were affected by purifying selection. The enrichment of trinucleotide pSTRs in coding sequences (CDS), 5′UTR and H3K4me3 regions suggests that trinucleotide STRs serve as important components in the exons and promoters of the corresponding genes. We demonstrated that, compared to SNPs, pSTRs provide comparable or even greater accuracy in determining the breed identity of individuals. We identified pSTRs that showed significant population differentiation between domestic pigs and wild boars in Asia and Europe. We also observed that some pSTRs were significantly associated with environmental variables, such as average annual temperature or altitude of the originating sites of Chinese indigenous breeds, among which we identified loss-of-function and/or expanded STRs overlapping with genes such as *AHR*, *LAS1L* and *PDK1*. Finally, our results revealed that several pSTRs show stronger signals in domestic pig—wild boar differentiation or association with the analysed environmental variables than the flanking SNPs within a 100-kb window.

**Conclusions:**

This study provides a genome-wide high-density map of pSTRs in diverse pig populations based on genome sequencing data, enabling a more comprehensive characterization of their roles in evolutionary and environmental adaptation.

**Supplementary Information:**

The online version contains supplementary material available at 10.1186/s12711-021-00631-4.

## Background

Short tandem repeats (STRs), also known as microsatellite DNA loci, are defined as tandem repetitive DNA elements with core unit lengths of one to six base pairs. STRs are largely present in the genomes of plants and animals [1], and they account for 3 and 3.4% of the human and mouse genomes, respectively [2]. STRs stem mainly from DNA polymerase slippage events during DNA replication [3]. In humans, the mutation rate of STRs ranges from 10^–8^ to 10^–2^ per locus per generation [4], which is much higher than the mutation rate of single nucleotide polymorphisms (SNPs) (10^–9^ to 10^–8^) [5]. Owing to their high genotype diversity and broad distribution across the genome, STRs have been widely used in genetic mapping of complex diseases [6], forensics [7], population genetics [8], linkage analysis [9], and cell line identification [10]. In humans, over 40 Mendelian diseases are caused by STRs, many of which are trinucleotide repeats. These diseases include the fragile X syndrome, which is associated with amplification of the CGG motif in the 5′ UTR of the *FMR1* gene [11], and Huntington’s disease, which is linked to CAG repeat expansion in the first exon of the *HTT* gene [12]. Studies have also shown important roles for STRs in the regulation of gene expression traits [13, 14].

Population genetics studies using dozens to hundreds of STRs have been carried out since the early 1990s [15]. Recently developed software tools such as lobSTR [16], STR-FM [17] and HipSTR [18] have enabled accurate genotyping of STRs from genome sequence data and their characterization in terms of their association with evolutionary and environmental adaptations and with complex traits in cohorts of various species including humans [19], macaques [20], cattle [21] and ducks [22]. For example, Xu et al. [21] generated the first high-density bovine STR map and identified STRs associated with dairy traits. Fan and his colleagues [22] found that STRs that differentiated Pekin ducks from mallards are associated with energy metabolism and nervous system genes. Forman et al. [23] observed that a (GAA)n repeat expansion in intron 35 of the *ITPR1* gene potentially causes spinocerebellar ataxia in Italian spinone dogs.

Pigs are among the most important domestic animals. To date, most of the population genetics studies that have been performed in pigs have been based on SNPs called from high-density SNP arrays [24] or second-generation sequencing data [25, 26], or on a small number (from dozens to hundreds) of STRs genotyped with the Sanger sequencing technology [27]. A recent study identified approximately 16,000 high-quality polymorphic STRs in sequence data from 102 pigs by referring to the Sscrofa10.2 genome assembly and characterized their genetic diversity among different breeds [28]. However, this study included a limited number of samples and breeds, which restricted its ability to investigate the diversity of STRs and their evolutionary roles in pigs. Moreover, the reference genome of pigs has been updated to Sscrofa11.1, which is a much better assembly than Sscrofa10.2 [29]. Therefore, characterizing the STRs in larger cohorts and using the latest porcine reference genome would be helpful to better understand the genetic diversity, evolutionary and environmental adaptations of STRs in pigs.

**Fig. 1:**
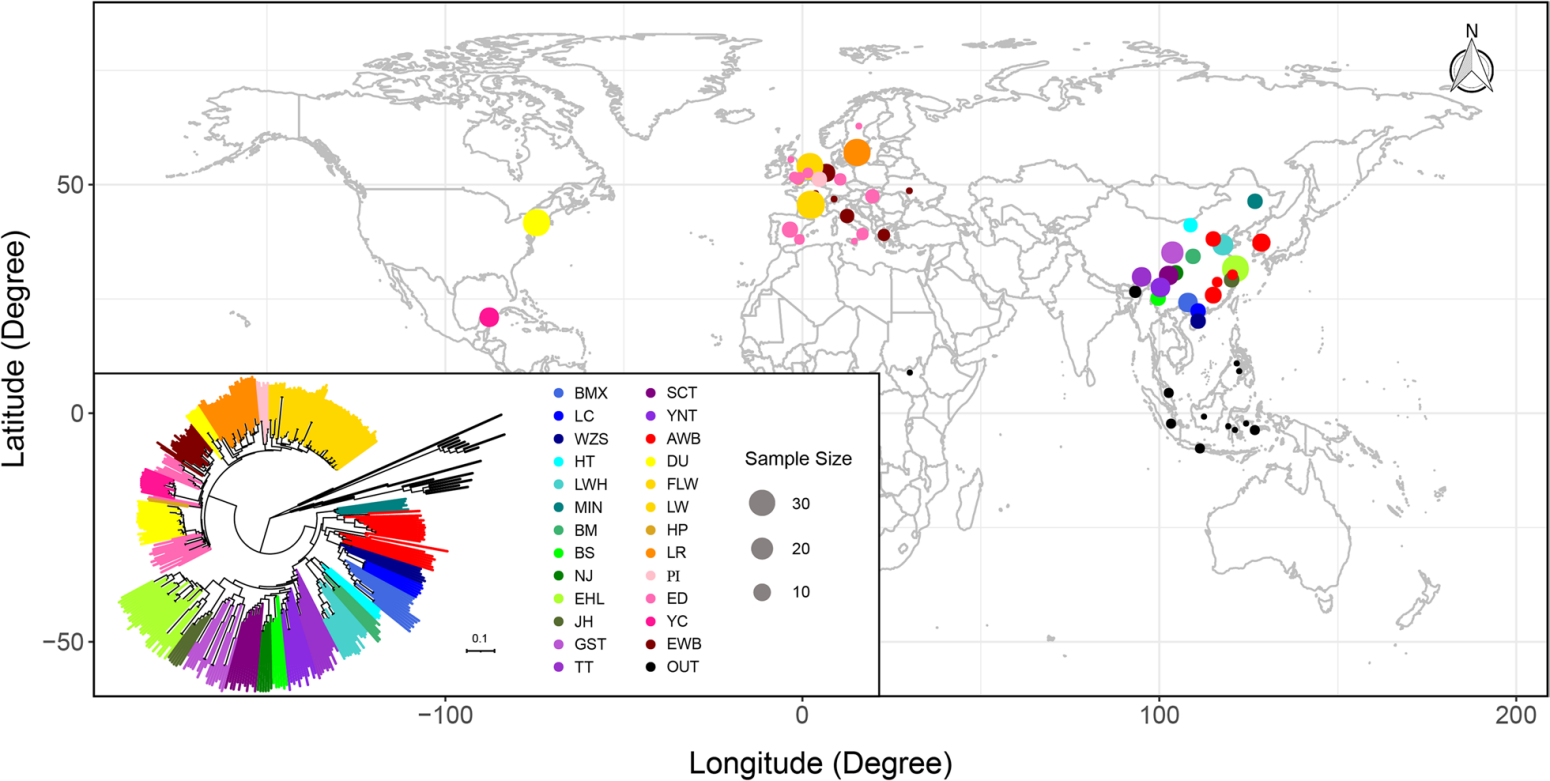
Geographical distributions and STR-based neighbor joining tree on 394 samples investigated in this study. The neighbor joining tree is constructed based on whole-genome STRs with the frequency of the major allele $\ge$ 0.05. Different colors indicate different breeds/populations, and the size of the dot indicates population size. The full names and abbreviations of the breeds/populations are as follows: *BMX* Bamaxiang, *LC* Luchuan, *WZS* Wuzhishan, *LWH* Laiwu, *HT* Hetao, *MIN* Min, *BM* Bamei, *BS* Baoshan, *NJ* Neijiang, *JH* Jinhua, *EHL* Erhualian, *YT* Yunnan Tibetan, *ST* Sichuan Tibetan, *GT* Gansu Tibetan, *TT* Tibet Tibetan, *AWB* Asian wild boars, *EWB* European wild boars, *ED* European domestic pigs, *DU* Duroc, *LR* Landrace, *PI* Pietrain, *HP* Hampshire, *LW* Large White, *FLW* French Large White, *YC* Yucatan, *OUT* Outgroups. Among them, BMX, LC, WZZ, LWH, HT, MIN, BM, BS, NJ, JH, EHL, YT, ST, GT and TT are Asian domestic pigs. DU, LR, PI HP, LW, FLW, YC are European commercial pigs

In this study, we investigated polymorphic STRs (pSTRs) in 330 domestic pigs from 22 indigenous pig populations, 24 Asian and 21 European wild boars, and 19 individuals from other Suidae species based on the Sscrofa11.1 genome assembly (Fig. 1). The identified pSTRs substantially augment the pSTRs landscape in pigs. We surveyed the pSTRs for their genomic distributions by different unit lengths, genetic diversities in different populations, and their accuracy in classifying individuals by breed identity. We annotated loss-of-function and expanded pSTRs. We also identified a number of pSTRs that showed significant differentiation between domesticated pigs and wild boars in Asia and Europe and pSTRs that are associated with environmental variables such as average annual temperature or altitude in Chinese indigenous pigs, and compared the signals of population differentiation or environmental adaptation-related pSTRs with those of SNPs called from the same dataset.

## Methods

### Samples

Sequence data of 394 pigs or other *Sus* species were used to genotype the genome-wide STRs, of which 241 were generated by our laboratory, and the rest were downloaded from public databases (see Additional file 1: Table S1 and Fig. 1). The 394 individuals comprise 24 Asian wild boars from South China and Korea; 157 Chinese domesticated pigs representing 15 indigenous breeds, including Bamaxiang (BMX), Luchuan (LC), Wuzhishan (WZS), Laiwu (LWH), Hetao (HT), Min (MIN), Bamei (BM), Baoshan (BS), Neijiang (NJ), Jinhua (JH), Erhualian (EHL), Yunnan Tibetan (YT), Sichuan Tibetan (ST), Gansu Tibetan (GT) and Tibet Tibetan (TT); 21 European wild boars; 43 European domesticated pigs; 130 international commercial pigs, including Duroc (DU), Landrace (LR), Pietrain (PI), Hampshire (HP) and Large White (LW) breeds, and 26 outgroup species, including seven *Sus cebifrons*, two *Sus verrucosus*, one *Sus celebensis*, one *Sus barbatus*, six *Porcula salvania*, one *Phacochoerus africanus* and one *Babyrousa babyrussa*.

### Annotation of repeat sequences in the reference genome

We downloaded the Sscrofa11.1 reference genome sequences from ENSEMBL (ftp://ftp.ensembl.org/pub/release-95/fasta/sus_scrofa/). The Tandem Repeat Finder (TRF v4.09) software [30] was used to search for potential STR loci in the reference genome with the following command: “${trf} genome.fa 2 7 7 80 10 20 6 -d–h”. All overlapping and equivocal STRs were excluded. RepeatMasker and RepeatModeler were used to detect other repeat elements, including short interspersed nucleotide elements (SINE), long interspersed nucleotide elements (LINE), long terminal repeat retrotransposons (LTR), and DNA transposable elements (DNA_TE). Briefly, RepeatMasker (v4.0.7) [31] was used to search for repeat sequences by BLAST search of the existing repeat database (Repbase v20170127) [32], while the de novo method in RepeatModeler (v1.0.11) [33] was applied to probe new repeat elements. Then, the results of the two programs were merged.

### Population-scale genotyping of STRs

For most individuals, the raw sequence data were filtered by removing pairs of reads in which one read contained more than 50% low quality (Q < 20) or less than 10% undetermined bases. The sequence reads from each sample were mapped to the Sscrofa11.1 genome assembly using the ‘mem’ algorithm in BWA v0.7.17-r1188 [34]. We used the default parameters of the lobSTR software to genotype STRs with motif lengths of 2 to 6 bp. The STRs supported by a minimum sequence depth of 5 (-loc-cov 5), and with quality scores higher than 0.8 (-loc-log-score 0.8), maximum reference lengths less than 80 bp (-loc-max-ref-length 80) and call rates higher than 60% (-loc-call-rate 0.6) were retained for further analyses. The STRs with more than two alleles were considered polymorphic STRs. The STR expansion score was defined as (95th percentiles of allele length—5th percentiles of allele length)/repeat unit length for each STR locus. STRs with expansion scores $\ge$ 10 were considered as expanded STRs. The STR dosage was defined as (i_*A*_ + i_*B*_) − 2r, where r and i are the lengths of the STR alleles of the reference genome and sample genome, respectively, and *A* and *B* indicate the two alleles at the STR locus. The genotyping and quality control procedures for SNPs were previously reported [35].

### Principal component analysis and phylogenetic analysis

Principal component analysis (PCA) of the 394 samples was performed using the EIGENSOFT/smartPCA software (v6.1.4) [36]. Briefly, for a specific STR locus with a number of alleles equal to n, we converted the individual genotypes into an indicator matrix with (n − 1) columns as input for the smartPCA program to generate eigenvectors and eigenvalues; a PCA figure was drawn using the R program. A neighbour-joining tree was built based on standard genetic distance using MEGA (v7) [37], and the figure was edited using iTOL [38]. In the stepwise mutation model, the genetic distance was estimated as: $D_{xy} = \frac{1}{4N}\mathop \sum \nolimits_{1}^{N} \mathop \sum \nolimits_{{i,j \in c\left( {1,2} \right)}} \left| {C_{{{\text{A}}i}} - C_{{{\text{B}}j}} } \right|$, where $C_{{{\text{A}}i}}$ is the repeat number of allele $i$ of individual ${\text{A}}$, and $N$ is the number of loci.

### Detection of signatures of population differentiation

The fixation index (${\text{R}}_{{{\text{st}}}}$) [39] is defined as: ${\text{R}}_{{{\text{st}}}} = \frac{{S_{t} - S_{w} }}{{S_{t} }}$, where $S_{w}$ is the average sum of squares of the differences in allele length within each population, and $S_{t}$ is the average sum of the squares of the differences in allele length across all individuals under investigation. ${\text{R}}_{{{\text{st}}}}$ values were Z-transformed. The top 5‰ STRs with the highest ${\text{R}}_{{{\text{st}}}}$ values are considered as significant. We combined significant STRs located within 5 kb of each other to obtain candidate differentiated regions (CDR), and the candidate genes located within a CDR were examined and discussed. Gene ontology (GO) and Kyoto Encyclopedia of Genes and Genomes (KEGG) analyses within CDR were performed using the ClusterProfiler package in R [40].

### Association with environmental variables using the general linear model (GLM)

The latitude and longitude coordinates of each population were assigned according to the locations where the samples were collected. We acquired bioclimatic data (mean annual temperature) from WorldClim 2.0 (www.worldclim.org; bioclimatic variables at 10 arc-minutes resolution) based on these coordinates. Altitude information was collected from ‘Animal Genetic Resources in China: Pigs’ [41]. An ordinary linear model was used to regress the two environmental variables (annual mean temperature and altitude) on the dosages of ~ 0.3 million high-quality STR loci using the R function lm() using STR data from 157 Chinese indigenous pigs with the first three principal components included as covariates. The significance threshold of the association was determined by the Bonferroni correction approach.

### Annotations of active promoters and enhancers using ChIP-Seq data

The histone modification markers, H3K4me3 and H3K27ac, indicate respectively active promoters, and active promoters and enhancers, which play fundamental roles in the regulation of gene expression [42]. To investigate the distribution of STRs in these regulatory elements, we downloaded H3K4me3 and K3K27ac ChIP-Seq data for liver samples from three pigs under ENA accession number PRJEB6906 [42]. Each sequencing data record was mapped to Sscrofa 11.1 using the ‘mem’ procedure in BWA v0.7.17-r1188 [34], and MACS [43] was used to detect H3K27ac peaks. For each histone marker, we merged the peaks identified in all individuals using the bedtools software and kept the 68,495 H3K27ac and 15,196 H3K4me3 merged peaks that were present in all three samples for further analysis. Fold enrichment analysis of different types of STRs in various genomic features was performed using the chi-squared test through the chisq.test function in R.

## Results

### Genotyping STRs in pig populations

In total, 2.8 million STRs were identified in the reference genome (Sscrofa 11.1), which largely outnumber other types of repetitive sequences. These 2.8 million STRs covered 2.23% of the reference genome (see Additional file 2: Figure S1). Since mononucleotide STRs are difficult to infer accurately, we excluded them from further analysis and kept 1.71 million STRs with 2 to 6 bp repeats as reference set in subsequent analyses. We were able to infer the genotypes of 1.68 million STRs in the 394 samples with average sequence depths of 21.6× (range from 5.0× to 45.7×) (see Additional file 1: Table S1). We identified an average of 1,556,291 STRs per individual (see Additional file 3: Figure S2a). The call rate of STRs increased with sequence depth and approached 90% in individuals with sequence depths higher than 18× (see Additional file 3: Figure S2b). Among the 394 samples, 246 and 66 were sequenced with 100-bp and 150-bp paired-end reads, respectively. We compared 100-bp and 150-bp paired-end sequencing strategies for their call rate on STRs with different reference allele lengths (see Additional file 4: Figure S3a). For STRs with reference alleles shorter than 60 bp, which accounted for more than 99% of the STRs under investigation, the two sequencing strategies gave very similar call rates (see Additional file 4: Figure S3b), which indicated that most of the polymorphic STRs investigated in this study would not be biased by the sequence read length.

To evaluate the accuracy of STR genotyping, we assessed the genotype concordance rate of STRs in 16 pairs of replicated samples from a heterogeneous pig population that were sequenced at low-depth (~ 7×) [44], and obtained an average concordance rate of 97.6% (see Additional file 5: Table S2). The rate of inconsistency in both alleles of the tested loci was less than 0.1%, suggesting that most genotyping errors were caused by allelic dropout. In addition, we compared STRs genotyped by lobSTR with those genotyped by HipSTR using sequence data from 61 selected pigs (see Additional file 1: Table S1). In total, 15,447,865 alleles from 290,147 STRs were present in the both lobSTR and HipSTR sets, among which, 14,415,224 (93.3%) were concordant and only 93,186 (0.6%) were inconsistent between the two datasets. The correlation of allelic dosages between HipSTR and lobSTR call sets was high (goodness-of-fit, R^2^ = 0.91) (see Additional file 6: Figure S4). In summary, this evidence supports the reliability of the STR genotypes profiled in this study.

### Characterization of polymorphic STRs

We identified 878,967 polymorphic STRs with at least two alleles segregating in the cohort, including 237,296 dinucleotide, 105,365 trinucleotide, 270,581 tetranucleotide, 149,351 pentanucleotide and 116,374 hexanucleotide STRs (see Additional file 7: Table S3), which taken together account for 0.69% of the reference genome sequence. The pSTRs identified here represent an increase of over 20% compared to the number identified in a previous study [28] (878,967 vs. 630,906).

**Fig. 2:**
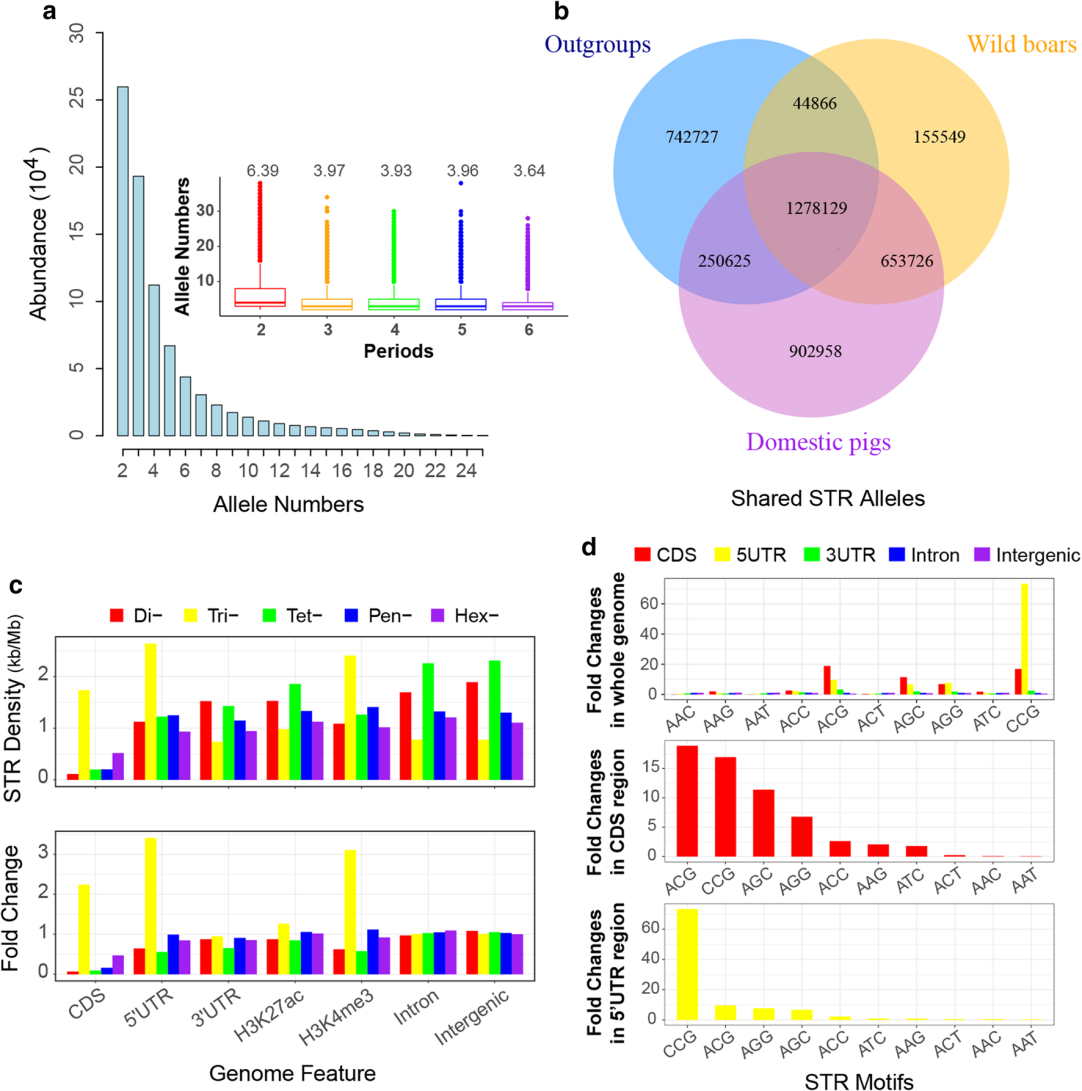
Characterization of polymorphic STRs in the pig genome. **a** Distribution of allele number of polymorphic STRs (pSTRs), inner density plot shows the distribution of the number of alleles of pSTRs by their repeat unit lengths. **b** Venn plot showing shared and specific pSTR alleles among outgroups, wild boar, and domesticated pigs. **c** Enrichment of polymorphic STRs in different genomic feature regions; the upper and bottom bar plots show the density distribution and fold-enrichment of different types of pSTRs in different genomic feature regions, respectively. Di-, Tri-, Tet-, Pen-, and Hex- denote dinucleotide, trinucleotide, tetranucleotide, pentanucleotide, hexanucleotide pSTRs, respectively. **d** Fold-enrichment of different types of trinucleotide pSTRs in different genomic feature regions (top plot), and specifically in coding (middle plot) and 5′ UTR (bottom plot) regions

We examined the frequencies of different motifs of pSTRs by repeat length and found that adenine rich motifs accounted for most (78.9%) pSTRs with two to five repeats (see Additional file 7: Table S3). For hexanucleotide pSTRs, ACAGCC is the most abundant motif, followed by AAAAAC, AAAAAG and AAAAAT. Further analysis found that ACAGCC is enriched in short interspersed nuclear elements (SINE, Odd Ratio = 3.50, chi-square test *P* < 2.2 × 10^–16^), reflecting that the ACAGCC repeat is an important component of SINE elements. The number of alleles per locus ranged from 2 to 38 (mean = 4.6 and median = 3), and only 116 (0.01%) loci had more than 30 alleles (Fig. 2a). Dinucleotide (mean = 6.39) and hexanucleotide (mean = 3.64) STRs have the largest and smallest average number of alleles, respectively, while the other types of STRs have a similar number of alleles (mean: 3.93 to 3.97) (Fig. 2a).

Next, we investigated the distribution of all STR alleles in outgroup species, and wild and domestic pigs. Out of the 4,028,579 alleles at the 878,967 pSTRs, 1,712,233 (42.50%) were specific to *Sus* scrofa, among which 902,958 were specifically identified in domestic pigs. In total, 1,278,129 (31.73%) alleles were shared across the outgroup species, wild boars and domestic pigs, and are thus considered ancestral or ancient STR alleles (Fig. 2b).

Then, we surveyed the genomic distribution of pSTRs. We computed the number of pSTRs in consecutive 1-Mb windows on the genome and defined windows with a number of pSTRs exceeding two standard deviations from the mean value as STR hot-spot regions. Based on this criterion, we identified pSTR hot spots on chromosomes 3, 6, 7, 10, 12, 14 and 17 (see Additional file 8: Figure S5 and Additional file 9: Table S4). It should be noted that the density of pSTRs on chromosome Y is extremely low, which could be due to the low genetic variability of the Y chromosome and poor assembly of this chromosome. While most of the pSTRs were located in intergenic and intronic regions, we identified 4595, 12,196, 6605 and 38,999 pSTRs located in coding, UTR, H3K4me3 and H3K27ac regions, respectively (Fig. 2c).

We compared the density of di-, tri-, tetra-, penta- and hexanucleotide STRs in different genomic features and found that the trinucleotide STRs were enriched approximately twofold in exons and approximately threefold in 5′UTR and H3K4me3 regions (Fig. 2c). Further examination of different trinucleotide repeat motif sequences revealed that ACG (fold change = 17.61), CCG (fold change = 16.09), AGC (fold change = 9.68) and AGG (fold change = 5.70) were among the most enriched motifs in the coding sequence (CDS) regions, while CCG showed extreme overrepresentation (fold change = 69.68) in the 5′UTR regions (Fig. 2d). Gene Ontology analyses showed that genes with coding regions containing a trinucleotide STR were enriched for genes related to DNA and RNA binding (*P* = 1.0 × 10^–8^), transcription factor binding (*P* = 5.3 × 10^–6^) and regulation of mRNA metabolism and gene expression (*P* = 7.6 × 10^–8^) (see Additional file 10: Figure S6a), whereas the genes harboring the trinucleotide STR in their 5′UTR were enriched in biological processes including cytoskeleton organization (*P* = 3.9 × 10^–5^), cellular senescence (*P* = 1.1 × 10^–4^) and protein kinase binding (*P* = 2.1 × 10^–4^) (see Additional file 10: Figure S6b). These results reflect a relaxation of negative selection on the trinucleotide repeats in the CDS and 5′UTR compared to the other types of STRs, and suggest that trinucleotides could constitute important components in both coding and promoter regions of genes. The functional relevance of trinucleotide repeats is also supported by studies in humans. For example, one study suggested that trinucleotide repeats encoding homopeptides can serve as important functional domains in different transcription factors [45], while trinucleotide repeats are also evidenced to be enriched in 5′UTR regions [46].

Compared to the trinucleotide pSTRs, the nontrinucleotide pSTRs, including di-, tetra-, penta- and hexanucleotide repeats, were underrepresented in coding regions (Fig. 2c), reflecting the effects of purifying selection. The higher fold enrichment of hexanucleotide repeats than the other non-trinucleotide repeats in coding regions could be explained by the fact that the strength of purifying selection on the hexanucleotide pSTRs in the CDS region was weaker than that on the other non-trinucleotide pSTRs, as they do not cause a frameshift of the protein sequences. Moreover, we also observed moderate underrepresentation of di- and tetra- nucleotide pSTRs in the promoter regions, which could reflect the effect of purifying selection represented by their disruptive effects on the regulatory function of promoters.

We examined the density of pSTRs with different repeats according to their distances from gene transcription start sites (TSS), and found a contrasting pattern with trinucleotide STRs being enriched around TSS ± 5 kb regions, and non-trinucleotide pSTRs being underrepresented in the regions around TSS (see Additional file 11: Figure S7), which is consistent with the results presented in Fig. 2c. Similar results were also observed in humans [46, 47]. Another trend is that the pSTR density is higher in the upstream region than in the downstream region of the TSS, which could be driven by purifying selection on the pSTRs located within the gene (see Additional file 11: Figure S7).

Furthermore, we compared the numbers of alleles, major allele frequencies (MAF) and polymorphic information contents (PIC) in the CDS, 5′UTR, H3K4me3 and intergenic regions (see Additional file 12: Figure S8). For all types of pSTRs, a consistent trend was observed with the average allele number and PIC being greatest in the intergenic regions, followed by the 5′UTR and H3K4me3, and lowest in the CDS regions. The significantly smaller number of alleles and smaller PIC values in the 5′UTR, H3K4me3 and CDS regions than in the intergenic regions could be attributed to purifying selection, which appeared to be stronger in CDS than in promoter regions.

Nucleotide variations in the coding region may directly affect gene structure and function. We identified 8989 loss of function (LOF) alleles for 5583 of the 0.87 million pSTR loci using the Ensembl Variant Effect Predictor software (VEP) [48], including frameshift mutations (33.06%), inframe variants (38.73%), splicing region variants (22.58%), coding sequence variants (4.26%) and start or stop codon variants (1.37%). These LOF variants intersected with 4302 genes and 9834 transcripts (see Additional file 13: Table S5). The genetic diversity of LOF pSTR loci was significantly lower than that of the other STRs (see Additional file 14: Figure S9a), reflecting the effects of purifying selection on the LOF pSTR loci. We examined the distribution of these LOF variants in different populations, and most of the LOF variants were found in only one or two populations, which suggests that the mutation occurred recently (see Additional file 14: Figure S9b).

### Population genetic analysis using genome-wide pSTRs

Principal component analysis (PCA) based on pSTRs clearly differentiated Asian from Western populations, and most of the individuals from the same populations were clustered together (see Additional file 15: Figure S10). To compare the accuracy of SNPs and STRs in classifying individuals by their breed/population identity, we performed phylogenetic analyses on 333 individuals from 25 populations (each population with at least five samples) based on STR and SNP genotypes, which showed that both STRs and SNPs can accurately classify most of the individuals by their breed/population memberships (see Additional file 16: Figure S11) and Fig. 1. In particular, the phylogenetic tree based on distance statistics (Dxy) accounting for STR repeat numbers seemed to perform even better than the phylogenetic tree based on the genome-wide SNP data. For example, the Hetao (HT) pigs were clustered together based on the distance that considers the copy number of core STR units, whereas this breed was divided into two branches on the phylogenetic tree based on SNPs, supporting the good informativeness of pSTRs in sample classification.

**Fig. 3:**
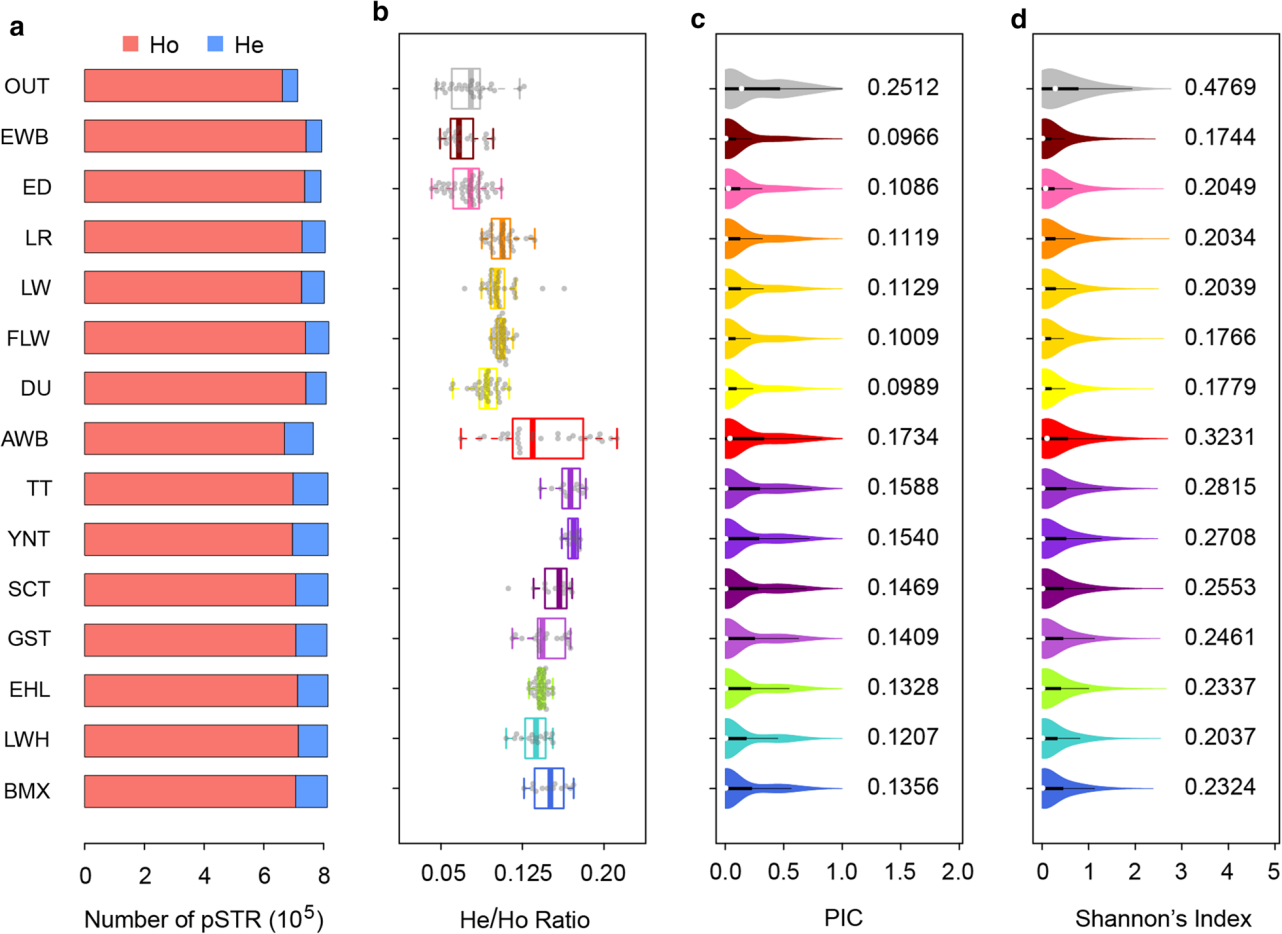
Genetic diversity of polymorphic STRs (pSTRs) in 15 populations with at least 12 individuals. The plots show the distributions of the **a** average number of heterozygotes (He) and homozygotes (Ho), **b** individual heterozygote/homozygote ratio, **c** Polymorphism Information Contents (PIC), and **d** Shannon’s index of pSTRs in the 15 populations. The analysis includes seven Asian domesticated breeds (*BMX* Bamaxiang, *LWH* Laiwu, *EHL* Erhualian, *YT* Yunnan Tibetan, *ST* Sichuan Tibetan, *GT* Gansu Tibetan, *TT* Tibet Tibetan); *AWB* Asian wild boars, *EWB* European wild boars,* ED* European domestic pigs; 4 Commercial breeds (*DU* Duroc, *LR* Landrace, *LW* Large White, *FLW* French Large White); and *OUT* Outgroups

Next, we examined the genetic diversity of each population based on different statistics calculated from pSTR genotypes (Fig. 3a–d), which included the number of polymorphic sites per breed/population, the ratio of heterozygous to homozygous STR sites per sample (He/Ho ratio), polymorphic information content (PIC) and Shannon’s index (H′). The results for the four statistics generally agree with each other, i.e. that the Asian pigs have a higher genetic diversity compared to that of the European pigs, for both domesticated pigs and wild boars. Other notable observations include the following: (1) Asian wild boars display a large variance in He/Ho ratio; (2) European wild boars have an even lower genetic diversity than domestic pigs; (3) among the Chinese domestic pigs, the Tibetan pigs have a higher genetic diversity on average than the other breeds; and (4) among the European pigs, LR and LW have a higher genetic diversity than the other breeds.

### STR expansion analysis

**Fig. 4:**
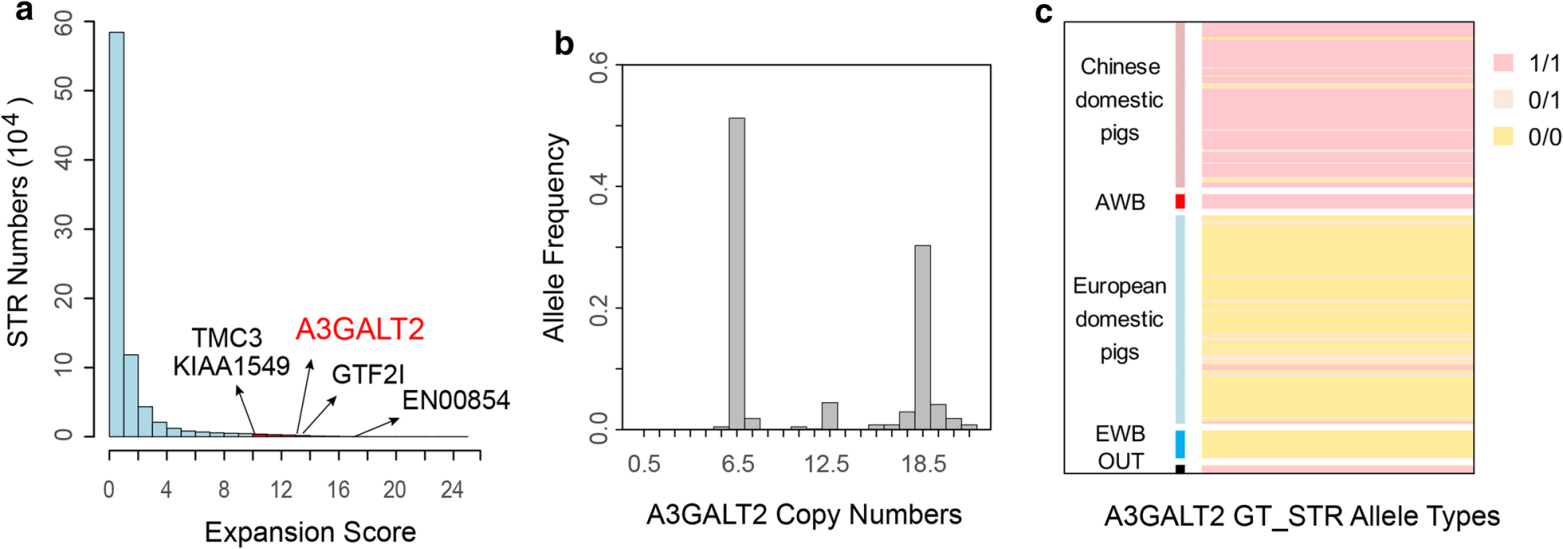
Analysis of polymorphic STR (pSTR) expansions. **a** Bar plot showing the distribution of expansion score, the genes corresponding to the four loss-of-function expanded pSTRs are highlighted and indicated by arrows. **b** Distribution of the copy number of alleles at the expanded pSTRs in the *A3GALT2* gene. **c** Heatmap displaying the distribution of genotypes of expanded/un-expanded (1/0) alleles in domestic pig and wild boar populations in Asia and Europe, where expanded/un-expanded alleles are defined as those with repeat numbers > / < 12.5

STR expansions have been identified as causal mutations in a number of human diseases [49]. Thus, we investigated STR expansion in our dataset according to a previously described approach (see “Methods”). In total, 14,217 (1.62%) of 878,967 loci were categorized as expanded STRs, including 14,074 dinucleotide, 76 trinucleotide, 65 tetranucleotide, one pentanucleotide and one hexanucleotide STRs (see “Methods” and Fig. 4a). Most of the expanded STRs were located in intergenic regions (n = 9593), followed by the intronic (n = 5472), noncoding (n = 176), UTR (n = 123) and CDS regions (n = 3). Among these, five were annotated as LOF STRs, including those on chr3:11,804,801–11,804,816 (*GTF2I*), chr6:89,562,510–89,562,522 (*A3GALT2*), chr7:50,149,248–50,149,277 (*TMC3*), chr5:80,787,071–80,787,090 (*ENSSSCG00854*), and chr18:10,785,011–10,785,038 (*KIAA1549*), all with relatively large expansion scores (Fig. 4a). Notably, we found that the expanded STR alleles in the *A3GALT2* gene showed extremely divergent frequencies in Chinese and European pigs (Fig. 4b, c). The STR variant was predicted to affect copy number of Thr-His repeats in the *A3GALT2* gene, which could ultimately alter the protein function. Since this repeat is located right at the beginning of the predicted A3GALT2 protein sequence, we compared the protein sequence of the porcine A3GALT2 (XP_020951514.1) with that of humans (NP_001073907) using BLASTP [50], and found that alignment starts at position 8 of the human protein and at position 27 of the pig protein sequence (see Additional file 17: Figure S12). In addition, there is a second methionine at position 18 of the porcine protein. In light of these analyses, we cannot rule out the possibility that the repeat could, in fact, be located in the 5′UTR of the *A3GALT2* gene. Furthermore, by searching the literature we found that *A3GALT2* is a homologue of the *ABO* gene, which belongs to the glycosyltransferase gene family [51]. The expression of this gene is generally low across various tissues except in the whole blood of humans according to the GTEx portal (www.gtexportal.org), which suggests that *A3GALT2* has a highly tissue-specific expression pattern; further investigation is required to validate the functional consequence of the expanded STRs in *A3GALT2*.

### Signatures of differentiation between domesticated pigs and wild boars in Asia and Europe

**Fig. 5:**
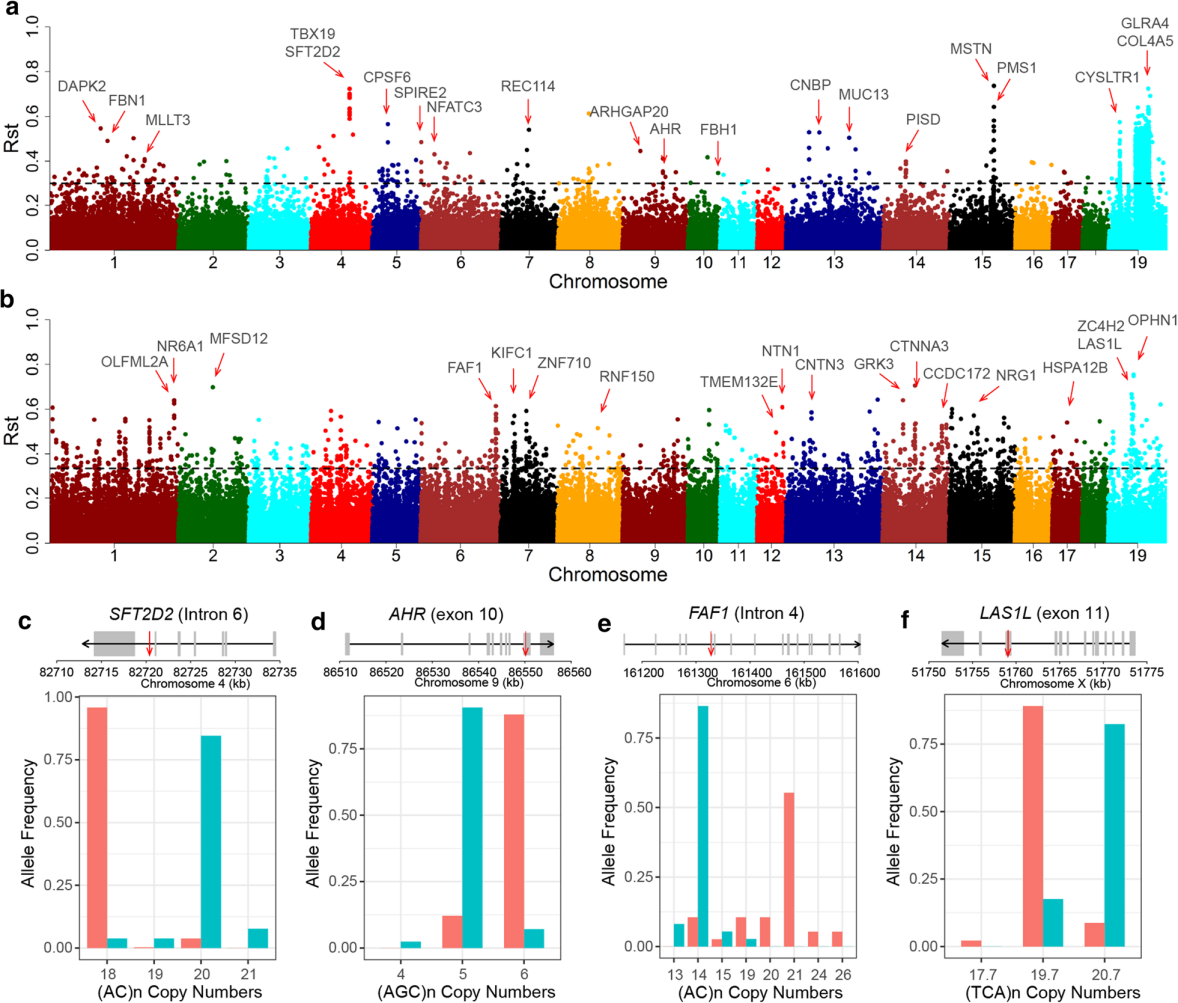
Population differentiation of pSTRs between domestic pig and wild boars in Asia and Europe. Manhattan plots showing the distributions of genome-wide ${\text{R}}_{{{\text{st}}}}$ values between domestic pigs and wild boars in Asia (**a**) and Europe (**b**). The genes intersecting with ± 5-kb flanking regions of the top STRs that show the highest ${\text{R}}_{{{\text{st}}}}$ values are indicated by red arrows. **c**, **d** Representative examples of distributions of copy numbers of pSTRs in Asian domestic pigs and wild boars in the *SFT2D2* (**c**) and *AHR* genes (**d**). **e**, **f** Representative examples of distributions of copy numbers of pSTRs in European domestic pigs and wild boars in the *FAF1* (**e**) and *LAS1L* genes (**f**). In **c**–**f**, the structure of the genes is plotted according to the GTF files from Ensembl (release 95) using customized R scripts. The black arrows in the genes indicate the transcripts that are encoded by the positive or negative strand of the genome. The red arrows indicate the location of STRs in the corresponding genes

To identify the STRs that underlie the phenotypic differentiation between domesticated pigs and wild boars, we computed the genome-wide fixation index (${\text{R}}_{{{\text{st}}}}$) between domesticated and wild pigs in Asia and Europe, separately. In Asia, the analysis was performed on 24 Chinese wild boars and 157 indigenous pigs, and revealed 1484 significantly differentiated pSTRs (Fig. 5a) and (see Additional file 18: Figure S13a, b and Additional file 19: Table S6), including one LOF STR located in the *AHR* gene and 18 expanded STRs (see Additional file 20: Table S7). We were able to assign 175 candidate genes to STRs located in the ± 5 kb flanking regions of genes. Among the top 20 candidate genes ranked by corresponding ${\text{R}}_{{{\text{st}}}}$ values, we found genes with functions related to organ development (*PMS1* [52]), puberty (*SFT2D2* [53], Fig. 5c), social behaviour (*GLRA4* [54], *TBX19* [35]) and fertility (*AHR* [35], Fig. 5d) and stature (*FBN1* [55]). One example is the (ACAATG)n repeat (chr4:82,723,830–82,723,847) located in intron 4 of the *SFT2D2* gene (${\text{R}}_{{{\text{st}}}}$ = 0.725, $Z_{Rst}$ = 21.84) (Fig. 5c), which is significantly differentially expressed between Bama Xiang pigs and commercial pigs and has been suggested to be related to sexual maturity [53]. The (AGC)n duplication in exon 2 of the *AHR* gene (Fig. 5d) has been reported to be associated with the control of reproductive traits in pig [35]. Moreover, we identified nine significant STRs with higher ${\text{R}}_{{{\text{st}}}}$ values than the signals of flanking SNPs within ± 100-kb regions (see Additional file 18: Figure S13c, d and Additional file 20: Table S7).

In the European group, 21 wild boars and 31 domesticated pigs were analysed. We identified 1325 significant STRs, including one LOF and 85 expanded STRs (see Additional file 20: Table S7). The 1325 significant STRs covered 479 coding genes, among which the genes that involve glutamatergic synapses (*P* = 2.44 × 10^–4^) were overrepresented (see Additional file 21: Table S8). Among the top differentiated STRs (Fig. 5b and (see Additional file 18: Figure S13d–f and Additional file 19: Table S6), we found genes with functions that are relevant to bone development (*NR6A1* [56] and *FAF1*), nervous system development (*OPHN1*, *ZC4H2*, *LAS1L*, *TMEM132E*, *CNTN3*, *NTN1*, *ITSN1*, and *NRG1*), signal transduction (*GRK3*) and coat colour (*MFSD12* [57]). Among these, we observed an expanded STR in intron 4 of the *FAF1* gene, where the wild boars showed an average of seven additional AC copies compared with domesticated pigs (Fig. 5e), and an in-frame deletion STR in exon 11 of *LAS1L*, for which domesticated pigs have on average one TCA copy less than wild boars (Fig. 5f), causing one aspartic acid less out of 20 aspartic acid repeats from the protein sequence. *LAS1L* has functions related to nervous system development, and mutations in this gene have been reported to cause mental retardation and neuromuscular disease [58]. Although most regions indicated by differentiated STRs were also indicated by SNP data in our study or in independent studies [59], we observed 84 STRs with stronger differentiation signals than SNPs within the same 100-kb region, of which eight were among the top 20 STRs showing the highest ${\text{R}}_{{{\text{st}}}}$ values (see Additional file 18: Figure S13a, b and Additional file 20: Table S7).

### Associations of pSTRs with local environmental adaptation in Chinese indigenous pigs

**Fig. 6:**
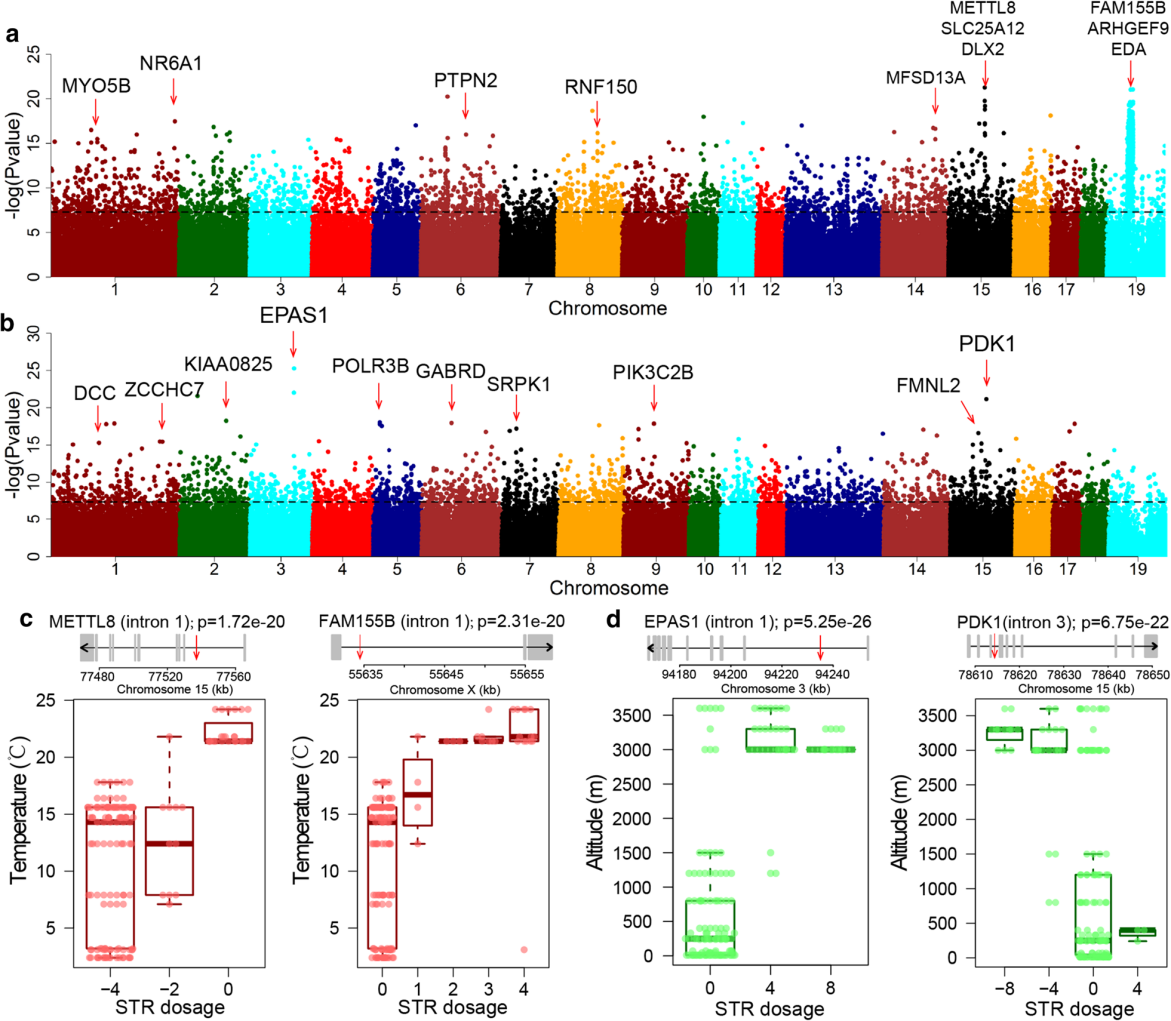
Associations of polymorphic STRs (pSTRs) with annual mean temperature and altitude among Chinese indigenous pigs. Manhattan plots of P values corresponding to the association of pSTRs with annual mean temperature (**a**) and altitude (**b**) of geographical regions where the indigenous breed originated. The genes intersecting with ± 5-kb flanking regions of the top STRs that show the highest ${\text{R}}_{{{\text{st}}}}$ values are indicated by red arrows. **c**, **d** Representative examples of distributions of STR dosages corresponding to the annual mean temperatures in the *METTL8* (**c**) and *FAM155B* genes (**d**). **e**, **f** Representative examples of distributions of STR dosages corresponding to the altitude values in the *EPAS1* (**e**) and *PDK1* genes (**f**). In **c**–**f**, the structure of the genes is plotted according to the GTF files from Ensembl (release 95) using customized R scripts. The black arrows in the genes indicate the transcripts that are encoded by the positive or negative strand of the genome. The red arrows indicate the location of STRs in the corresponding genes

Chinese indigenous pigs originate from diverse geographical regions with distinct environmental conditions. Temperature and altitude are two important environmental variables that vary broadly among the regions of China that are inhabited by pigs, e.g., the average annual temperature ranges from 24.2 °C in Hainan Province (home to Wuzhishan pigs) to 7.1 °C in Mongolia (home to Hetao pigs). Moreover, the Tibetan pigs are mainly distributed in the plateau areas of Sichuan, Gansu, Tibet and Yunnan. Their habitats have altitudes of 3000 to 4500 m, low oxygen, low temperature and high ultraviolet radiation. To identify STRs associated with the adaptation of Chinese indigenous pigs to local temperatures and altitudes, we implemented a generalized linear model to associate the 2.9 million STRs across the genome with the two environmental variables. With the Bonferroni correction threshold of *P* = 8.33 × 10^–8^, 3268 and 2692 pSTRs were significantly correlated with annual mean temperature and altitude, respectively (Fig. 6a, b).

Among the 3268 STRs that were significantly associated with annual mean temperature, most of them were in intergenic (69.2%) and intronic (34.9%) regions. Moreover, we found seven LOF and 88 expanded STRs (see Additional file 22: Table S9). The 3268 STRs encompassed 807 unique coding genes, which were enriched in the calcium signaling pathway (*P* = 4.04 × 10^–4^) and glutamatergic synapses (*P* = 4.05 × 10^–4^; see Additional file 23: Table S10). The most significantly associated STR regions contain genes with functions that are relevant to skeletal muscle tissue development (*METTL8*), calcium channel activity (*FAM155B*, *SLC25A12*), and hair follicle development (*EDA* [60]), which suggests that these genes are potentially involved in the adaptation to local temperatures (see Additional file 24: Table S11 and Fig. 6c). Other significantly correlated STRs were associated with congenital diarrheal disorders (*MYO5B* [61]), the insulin receptor signaling pathway (*PTPN2*), brain development (*DLX2*), and nervous system development (*ARHGEF9*). Furthermore, we found 482 STRs that were more significant than the SNPs in 100-kb flanking regions (see Additional file 25: Figure S14a, b and Additional file 23: Table S10). Nevertheless, since Northern and Southern pigs in China have an approximate split time of 0.6 million years [62], the local temperatures could have changed after the split of different indigenous pig breeds. Therefore, the associations should be interpreted with caution.

The 2692 altitude-associated STRs included four LOF and 47 expanded STRs (see Additional file 22: Table S9). The significance of the association of the four LOF STRs ranged from 6.2 × 10^–8^ to 1.2 × 10^–10^, which was much less significant than that of the STRs with the most significant signals. We assigned 742 genes to the 2692 STRs. These genes were enriched in axon guidance (*P* = 0.05) (see Additional file 23: Table S10). Notably, we observed that the three most strongly associated STR loci intersect with the *EPAS1* and *PDK1* genes (Fig. 6d). *EPAS1* is a well-known gene that is involved in adaptation to high altitudes in multiple species including humans, dogs and yaks [63–65]. We identified two STRs, including AAAC and AG repeats in introns 1 and 3 of *EPAS1*, respectively. Both STRs overlapped with the liver H3K27ac peaks. *PDK1* is related to response to hypoxia in cancer [66]. The other genes that intersect with the top associated pSTRs were related to immune system process (*POLR3B*), Akt pathway (*PIK3C2B*), nervous system development (*GABRD*, *DCC*) and mRNA processing (*SRPK1*, *ZCCHC7*). In addition, we found 306 STRs that were more significant than the SNPs within the 100 kb flanking region (see Additional file 25: Figure S14c, d and Additional file 23: Table S10).

## Discussion

We report the largest STR-based population genetics study to date that comprises 394 individuals from 22 domestic pig populations, wild boars from both Asia and Europe, and more than 20 outgroup species. We identified nearly 200 thousand more pSTRs than a previous study [28], thereby considerably expanding the landscape of pSTRs in pigs. The generated pSTR dataset provides an excellent resource to describe the genetic diversity and allelic frequency spectrum of STRs in diverse pig populations. We also demonstrated that pSTRs are informative for accurately determining the breed identity of individuals. Moreover, we showed several lines of evidence, including (1) underrepresentation of non-trinucleotide repeats in the promoter and coding regions of genes, (2) lower genetic diversity of STRs in coding than in intronic and intergenic regions and (3) significant associations of STRs with differentiation between wild boar and domesticated pigs and environmental variables, suggesting that pSTRs could be targets of negative or positive selection, i.e., could influence the fitness of individuals, which makes them worthy of inclusion in genetic mapping and population genetic studies.

We observed a remarkable enrichment of trinucleotide STRs in the 5′UTR (CCG) sequences and exons (ACG, CCG, AGC and AGG) of genes and an overrepresentation of the corresponding genes in biologically meaningful pathways or molecular functions, suggesting that trinucleotide STRs could be an important functional component of exons and promoters of the corresponding genes. Several other studies also support this assumption, e.g., a study in humans also observed the overrepresentation of CNG trinucleotide repeats in exons and proposed that the trinucleotide repeats are potential functional genetic elements [67]. Moreover, it was reported that AGG and AGC repeats could serve as binding motifs for some transcriptional or translation factors that regulate the expression and translation of target genes [68]. In addition, we observed that ACAGCC is the most abundant hexanucleotide motif and is enriched in short interspersed nuclear elements (SINE) in pig genomes, SINE being a class of transposable elements that play important roles in the evolution of various species [69]; thus, this result suggests a potential role of the ACAGCC repeats in the evolution of genomes.

We identified a series of pSTRs annotated as LOF and/or expanded loci showing significant signals of population differentiation or association with environmental variables. These include the in-frame CGA repeats in the *AHR* gene (potentially associated with fertility [35]) and TCA repeats in the *LAS1L* gene (neurodevelopment and behavior [58]) showed significant differentiation between domesticated and wild boars in Asia and Europe, respectively. The LOF STRs in the *HOXB3* gene (associated with angiogenesis [70]) is associated with annual mean temperature. In addition, expanded STRs in the *DENND1B*, *NCOA1*, *COL6A3* and *TFRC* genes were associated with altitude. Among them, *NCOA1* and *TFRC* are closely associated with HIF signaling pathways, which are related to oxygen metabolism [71, 72]. The LOF pSTRs could potentially affect the function of the corresponding gene and in turn alter the fitness of individuals, and STR expansions have also been proven to be responsible for several diseases in humans [73–75]. Therefore, LOF and/or expanded STRs and their corresponding genes could be strong candidates for follow-up functional studies.

We identified a number of STRs that showed significant differentiation between wild boars and domesticated pigs in China and Europe. Some regions including the *TBX19* [35], *NR6A1* [56], *OLFML2A* [76] and *AHR* [35] genes have been previously identified based on the SNP dataset. Notably, we also revealed a number of new signals based on STR data, including the STRs in the *GLRA4* gene, which differentiated Asian domesticated pigs from wild boars, and STR regions overlapping with the nervous system-related genes *OPHN1*, *ZC4H2* [77], *LAS1L* [58], *ITSN1* and *NRG1*, which showed significant differentiation between European domesticated pigs and wild boars. Similarly, in the association analysis with annual mean temperature and altitude, our findings recapitulated well-characterized regions for altitude adaptation, such as the *EPAS1* gene [64]. In addition, we found that in a number of regions the association signals of STRs were stronger than those of SNPs. These included STRs associated with annual mean temperature close to or in biologically sensible genes that are involved in hair follicle development (*EDA*, [60]), skeletal muscle development (*METTL8*), coagulation and accumulation of platelets (*PDIA4*, [78]), calcium signalling pathway (*FAM155B* and *SLC25A12*), and altitude associated STR regions overlapping with genes related to response to hypoxia (*PDK1* [66], *PIK3C2B* [79] and *PGR* [80]). Recently, several studies have observed the greater significance of STRs than of SNPs in associations with gene expression traits in humans [14, 81]. Overall, we suggest that STRs could provide complementary information to SNPs and Indels in population genetics studies that aim at identifying genome regions under artificial or environmental selection.

Nevertheless, due to the limitations of second-generation sequencing technology such as (1) biases introduced from the PCR amplification process, (2) low sensitivity and accuracy in regions with an abnormal GC content, and (3) short read lengths that cannot span large STR loci, the set of STRs revealed in this study may still be incomplete. The use of third-generation sequencing technology that results in much longer read lengths should be able to provide additional STRs that may have been missed in this study.

## Conclusions

Using next-generation sequencing data from 394 genomes of 22 domestic pig breeds, wild boars, and outgroups worldwide, we revealed 878,967 genomic polymorphism STRs (pSTRs), providing a catalogue of pSTRs with LOF variants and genotype expansion. We demonstrated that the density and allelic spectrum of pSTRs in exons were shaped by purifying selection. Moreover, we showed evidence that CCG, AGG, and AGC repeats were significantly enriched in CDS, 5′UTR, and promoter regions, and that ACAGCC repeats were abundant in SINE elements, which suggests that these STRs have important functions in the pig genome. We also found that a number of pSTRs have more significant signals than SNPs in analyses of differentiation between wild boars and domestic pigs and associations with environmental adaptability, supporting that STRs can provide complementary information for identifying selection signals in population genetics studies, and potential genetic mapping studies for complex traits, improving upon results based on SNPs. Overall, this study presents the most comprehensive catalogue of polymorphic STRs in pigs to date and illuminates the role of STRs in the evolution, domestication and environmental adaptation of pigs.

## Supplementary Information

**Additional file 1: Table S1.** Detailed information on the 394 samples investigated in this study.

**Additional file 2: Figure S1.** Genomic coverage and counts of short tandem repeats (STRs) and other repeat elements in Sus scrofa11.1. (**a**) and (**b**) shows the genome coverages and counts of different types of repeat elements, respectively, with STRs highlighted by black color. (**c**) and (**d**) genome coverages and counts of different types of STRs with unit lengths from 2 to 6.

**Additional file 3: Figure S2.** Short tandem repeat (STRs) calling in 394 samples. (a) Histogram showing distribution of number of STRs called from each of the 394 samples. (b) Scatter plot showing the call rate of STRs according to sequence depths. Different colors represent different breeds/populations. The full names and abbreviations of the breeds/populations are as follow: Bamaxiang (BMX), Luchuan (LC), Wuzhishan (WZS), Laiwu (LWH), Hetao (HT), Min (MIN), Bamei (BM), Baoshan (BS), Neijiang (NJ), Jinhua (JH), Erhualian (EHL), Yunnan Tibetan (YT), Sichuan Tibetan (ST), Gansu Tibetan (GT), Tibet Tibetan (TT), Asian wild boars (AWB), European wild boars (EWB), European domestic pigs (ED), Duroc (DU), Landrace (LR), Pietrain (PI), Hampshire (HP), Large White (LW), French Large White (FLW), Yucatan (YC) and Outgroups (OUT). Among them, BMX, LC, WZZ, LWH, HT, MIN, BM, BS, NJ, JH, EHL, YT, ST, GT and TT are Asian domestic pigs. DU, LR, PI HP, LW, FLW, YC are European commercial pigs.

**Additional file 4: Figure S3.** Effects of sequence read lengths and short tandem repeat (STRs) lengths on the call rate of STRs. (a) Reference allele length distribution of identified STRs. (b) Box plot showing the impacts of sequence read lengths (100 vs. 150 bp) and STRs lengths on call rates of STRs.

**Additional file 5: Table S2.** Comparison of 16 pairs of replicated samples for pSTRs calling.

**Additional file 6: Figure S4.** Consistency of repeat dosages inferred using HipSTRs and LobSTRs in 61 selected samples with average read depths between 23.5× and 35.7×. The size of the dots is proportionate to the counts of STRs.

**Additional file 7: Table S3.** Summary of different types of polymorphic short tandem repeats (pSTRs) by motif sequences and lengths.

**Additional file 8: Figure S5.** Distribution of different types of polymorphic short tandem repeats (STRs) by their unit lengths across the genome. The Manhattan plots show the distributions of the number of (a) Di-, (b) Tri-, (c) Tet, (d) Pen, (e) Hex STRs within 1-Mb sliding windows across the genome, the distribution of H3K4me3 (bp/Mb) (f), H3K27ac (bp/Mb) (g), GC content (bp/Mb) (h) and mean heterozygosity of the pSTRs in a 1 Mb sliding widows (i) were also included for comparison.

**Additional file 9: Table S4.** Summary of polymorphic short tandem repeats (pSTRs) hotspots on different chromosomes.

**Additional file 10: Figure S6.** Gene ontology enrichment analysis on genes harboring trinucleotide polymorphic short tandem repeats (pSTRs) in their (a) CDS and (b) 5′UTR regions. BP stands for biological process, CC for cellular component and MF for molecular function.

**Additional file 11: Figure S7.** Density of different types of polymorphic short tandem repeats (pSTRs) in ± 100 bp flanking region of transcriptional start sites (TSS) across the genome. Different colors denote different types of pSTRs with unit lengths from 2 to 6.

**Additional file 12: Figure S8.** Genetic diversities (measured by allele number and polymorphic information content) of different types of polymorphic short tandem repeats with unit lengths from 2 to 6 in 5′UTR, CDS, H3K4me3 and intergenic regions. The p-value was estimated using wilcox.test in ggpubr package in R program.

**Additional file 13: Table S5.** Summary of loss-of-function alleles in terms of their genomic positions, associated genes, types, allele sequences, allele frequencies and locations in gene bodies.

**Additional file 14: Figure S9.** Properties of loss-of-function (LOF) polymorphic short tandem repeats (pSTRs) alleles. (a) Comparison of expected heterozygosity between LOF pSTRs and the other pSTRs. (b) Frequency distribution of LOF pSTRs alleles shared between populations.

**Additional file 15: Figure S10.** Principal component analysis (PCA) based on genome-wide STRs using 394 samples. The full names and abbreviations of the breeds/populations are as follow: Bamaxiang (BMX), Luchuan (LC), Wuzhishan (WZS), Laiwu (LWH), Hetao (HT), Min (MIN), Bamei (BM), Baoshan (BS), Neijiang (NJ), Jinhua (JH), Erhualian (EHL), Yunnan Tibetan (YT), Sichuan Tibetan (ST), Gansu Tibetan (GT), Tibet Tibetan (TT), Asian wild boars (AWB), European wild boars (EWB), European domestic pigs (ED), Duroc (DU), Landrace (LR), Pietrain (PI), Hampshire (HP), Large White (LW), French Large White (FLW), Yucatan (YC) and Outgroups (OUT). Among them, BMX, LC, WZZ, LWH, HT, MIN, BM, BS, NJ, JH, EHL, YT, ST, GT and TT are Asian domestic pigs. DU, LR, PI HP, LW, FLW, YC are European commercial pigs.

**Additional file 16: Figure S11**. Genome-wide SNP and STRs clustering of multiple breeds using unsupervised clustering (method = complete). The colors show the breeds/populations to which the sample belongs. Only breeds with more than five samples were kept for analysis. The full names and abbreviations of the breeds/populations are as follow: Bamaxiang (BMX), Luchuan (LC), Wuzhishan (WZS), Laiwu (LWH), Hetao (HT), Min (MIN), Bamei (BM), Baoshan (BS), Neijiang (NJ), Jinhua (JH), Erhualian (EHL), Yunnan Tibetan (YT), Sichuan Tibetan (ST), Gansu Tibetan (GT), Tibet Tibetan (TT), Asian wild boars (AWB), European wild boars (EWB), European domestic pigs (ED), Duroc (DU), Landrace (LR), Pietrain (PI), Hampshire (HP), Large White (LW), French Large White (FLW), Yucatan (YC) and Outgroups (OUT). Among them, BMX, LC, WZZ, LWH, HT, MIN, BM, BS, NJ, JH, EHL, YT, ST, GT and TT are Asian domestic pigs. DU, LR, PI HP, LW, FLW, YC are European commercial pigs.

**Additional file 17: Figure S12.** Comparison of the human protein sequence (Query sequence, NP_001073907) with the porcine protein sequence (Subject Sequence, XP_020951514.1) using BLASTP.

**Additional file 18: Figure S13.** Regional signatures of population differentiation between domestic pig and wild boars in Asia (a) and Europe (b–d), where the STRs showed higher ${\text{R}}_{{{\text{st}}}}$ values than SNPs within ± 100 kb flanking regions.

**Additional file 19: Table S6.** Summary of candidate genes assigned to the most differentiated polymorphic short tandem repeats (pSTRs) between domestic and wild boars in Asia and Europe. The description of the function of these genes is based on the annotation entries of GO and KEGG.

**Additional file 20: Table S7.** Summary of polymorphic short tandem repeats (pSTRs) showing significant differentiation between domestic pigs and wild boars in Asia or Europe that were annotated as LOF pSTRs, expanded pSTRs, or showing greater differentiation than SNPs within ± 100 kb flanking regions.

**Additional file 21: Table S8.** KEGG and GO enrichment analysis on genes assigned to polymorphic short tandem repeats (pSTRs) that show significant differentiation between domestic and wild boars in Asia or Europe.

**Additional file 22: Table S9.** Summary of polymorphic short tandem repeats (pSTRs) showing significant associations with annual mean temperatures or altitudes and that were annotated as LOF pSTRs or expanded pSTRs, or showing greater differentiation than SNPs within ± 100 kb flanking regions.

**Additional file 23: Table S10.** KEGG and GO enrichment analysis on genes assigned to polymorphic short tandem repeats (pSTRs) that show significant associations with annual mean temperatures or altitudes among the Chinese indigenous breeds.

**Additional file 24: Table S11.** Summary of candidate genes assigned to top polymorphic short tandem repeats (pSTRs) with the most significant associations with annual mean temperatures or altitudes. The description of the function of these genes is based on the annotation entries of GO and KEGG.

**Additional file 25: Figure S14.** Regional plots of association signals of pSTRs with annual mean temperature (a, b) and altitude (c, d) among Chinese indigenous pig populations, for which STRs show stronger associations than SNPs within ± 100 kb flanking regions.

## Data Availability

Publicly available data analyzed in this study can be found at the NCBI Sequence Read Archive (https://www.ncbi.nlm.nih.gov/sra) under accession numbers PRJNA398176, PRJNA488327, PRJNA213179, PRJNA550237, PRJEB1683, PRJEB9922, PRJNA260763, PRJNA239399, PRJNA255085, PRJNA320525, PRJNA369600, PRJEB30129, and PRJEB9326. Scripts and datasets used in this study are available from https://github.com/jxlabWzZ/Susrepeats.
