# Supplementary figures and images for: A worldwide map of swine short tandem repeats and their associations with evolutionary and environmental adaptations
Source: Genet Sel Evol. 2021 Apr 23;53:39. doi: 10.1186/s12711-021-00631-4 (PMC8063339; doi:10.1186/s12711-021-00631-4)

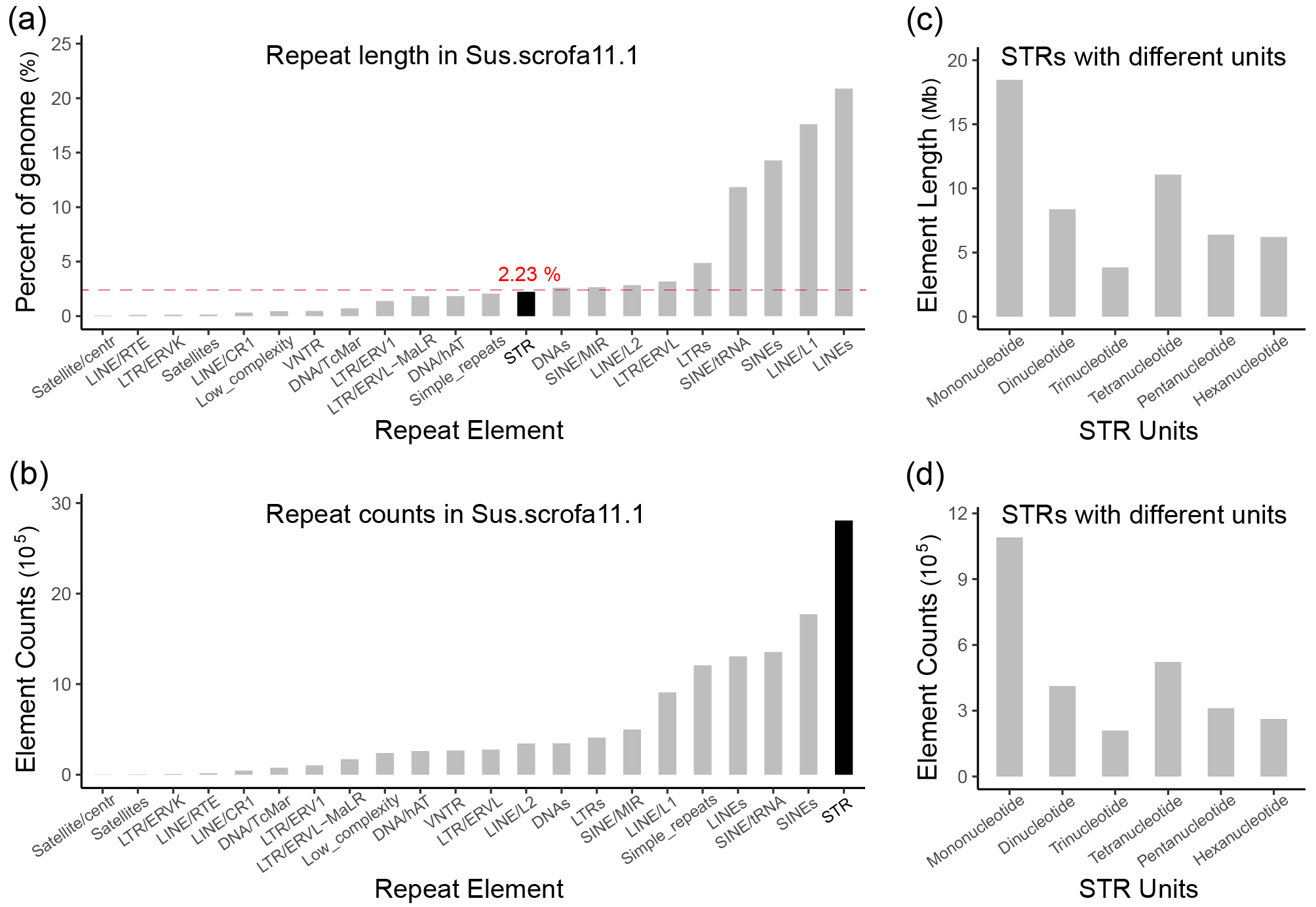

Supplement: Supplementary file 2 — Additional file 2: Figure S1. Genomic coverage and counts of short tandem repeats (STRs) and other repeat elements in Sus scrofa11.1. (a) and (b) shows the genome coverages and counts of different types of repeat elements, respectively, with STRs highlighted by black color. (c) and (d) genome coverages and counts of different types of STRs with unit lengths from 2 to 6. [file 12711_2021_631_MOESM2_ESM.png]

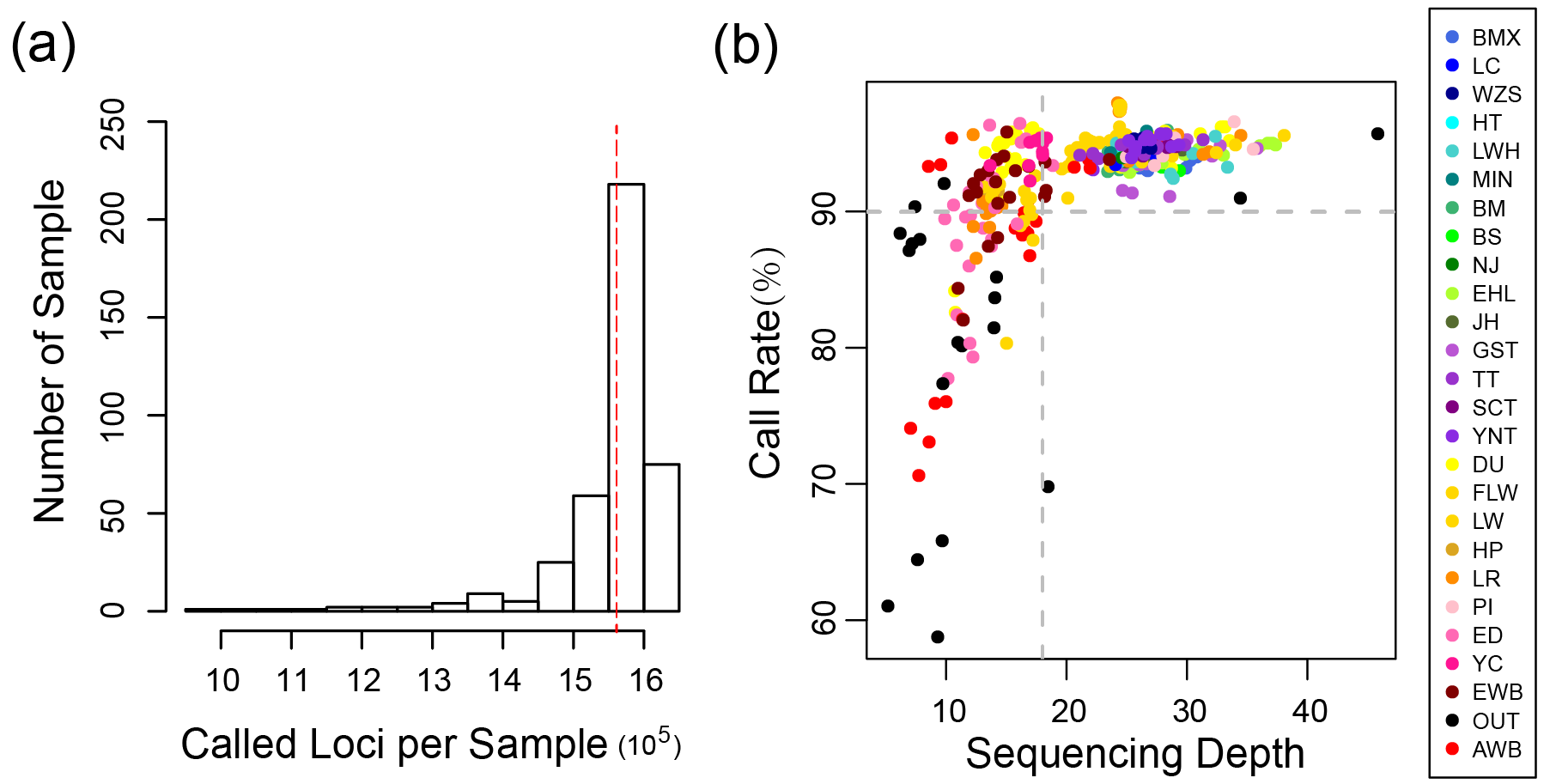

Supplement: Supplementary file 3 — Additional file 3: Figure S2. Short tandem repeat (STRs) calling in 394 samples. (a) Histogram showing distribution of number of STRs called from each of the 394 samples. (b) Scatter plot showing the call rate of STRs according to sequence depths. Different colors represent different breeds/populations. The full names and abbreviations of the breeds/populations are as follow: Bamaxiang (BMX), Luchuan (LC), Wuzhishan (WZS), Laiwu (LWH), Hetao (HT), Min (MIN), Bamei (BM), Baoshan (BS), Neijiang (NJ), Jinhua (JH), Erhualian (EHL), Yunnan Tibetan (YT), Sichuan Tibetan (ST), Gansu Tibetan (GT), Tibet Tibetan (TT), Asian wild boars (AWB), European wild boars (EWB), European domestic pigs (ED), Duroc (DU), Landrace (LR), Pietrain (PI), Hampshire (HP), Large White (LW), French Large White (FLW), Yucatan (YC) and Outgroups (OUT). Among them, BMX, LC, WZZ, LWH, HT, MIN, BM, BS, NJ, JH, EHL, YT, ST, GT and TT are Asian domestic pigs. DU, LR, PI HP, LW, FLW, YC are European commercial pigs. [file 12711_2021_631_MOESM3_ESM.png]

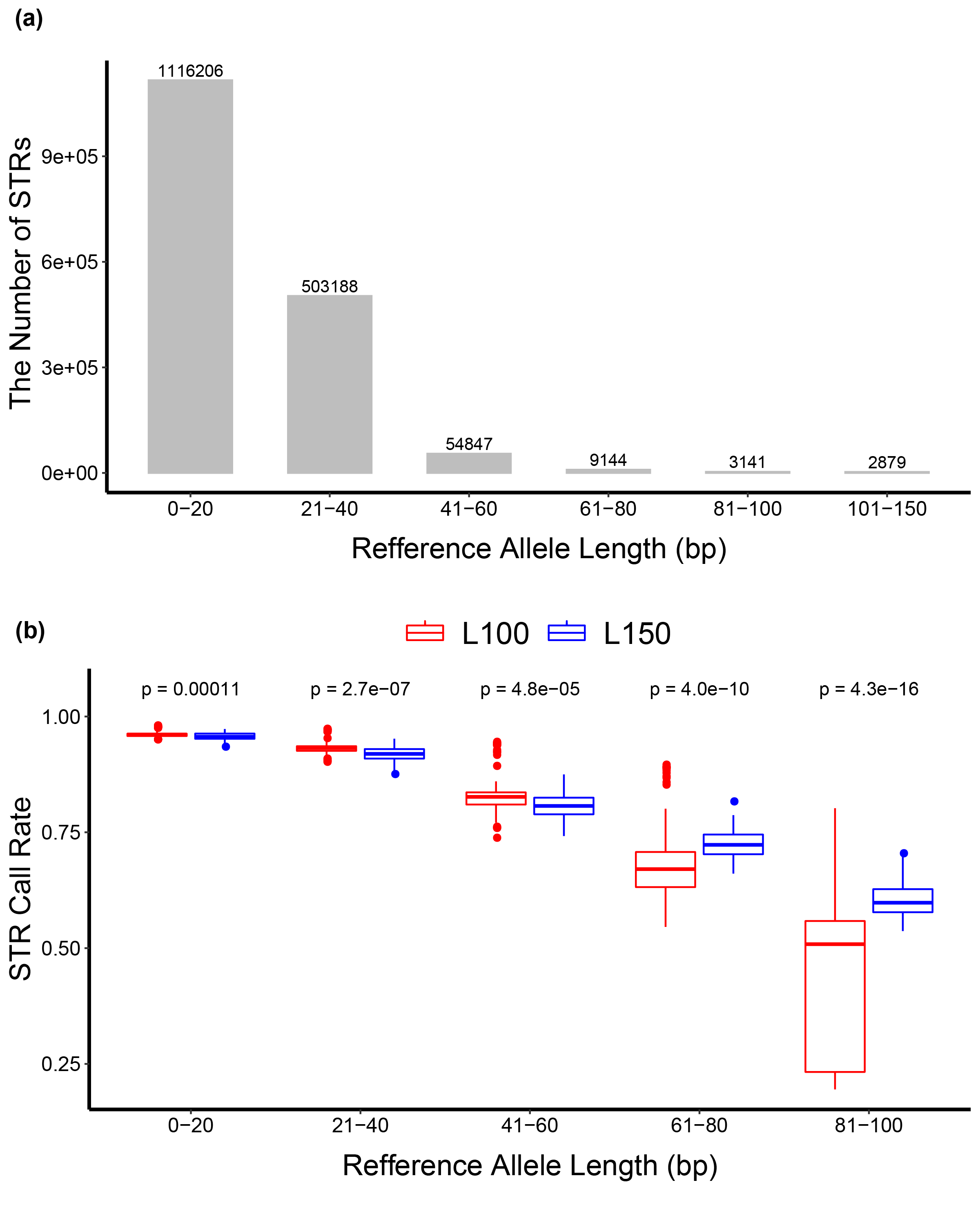

Supplement: Supplementary file 4 — Additional file 4: Figure S3. Effects of sequence read lengths and short tandem repeat (STRs) lengths on the call rate of STRs. (a) Reference allele length distribution of identified STRs. (b) Box plot showing the impacts of sequence read lengths (100 vs. 150 bp) and STRs lengths on call rates of STRs. [file 12711_2021_631_MOESM4_ESM.png]

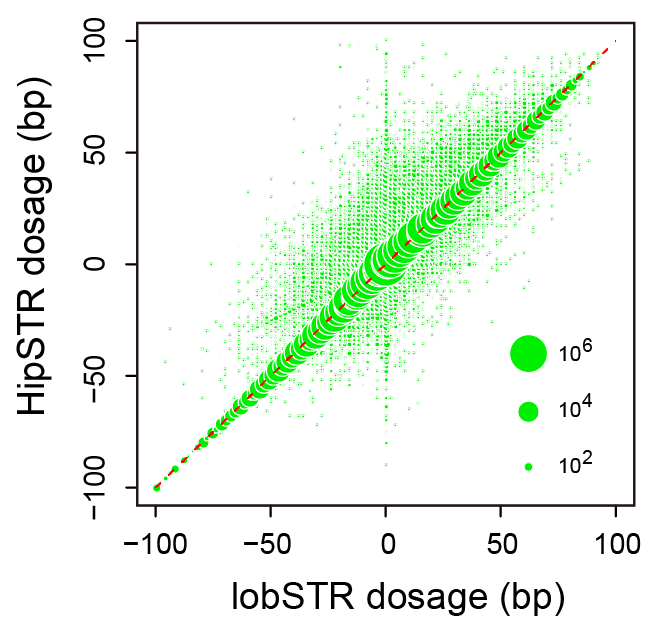

Supplement: Supplementary file 6 — Additional file 6: Figure S4. Consistency of repeat dosages inferred using HipSTRs and LobSTRs in 61 selected samples with average read depths between 23.5× and 35.7×. The size of the dots is proportionate to the counts of STRs. [file 12711_2021_631_MOESM6_ESM.png]

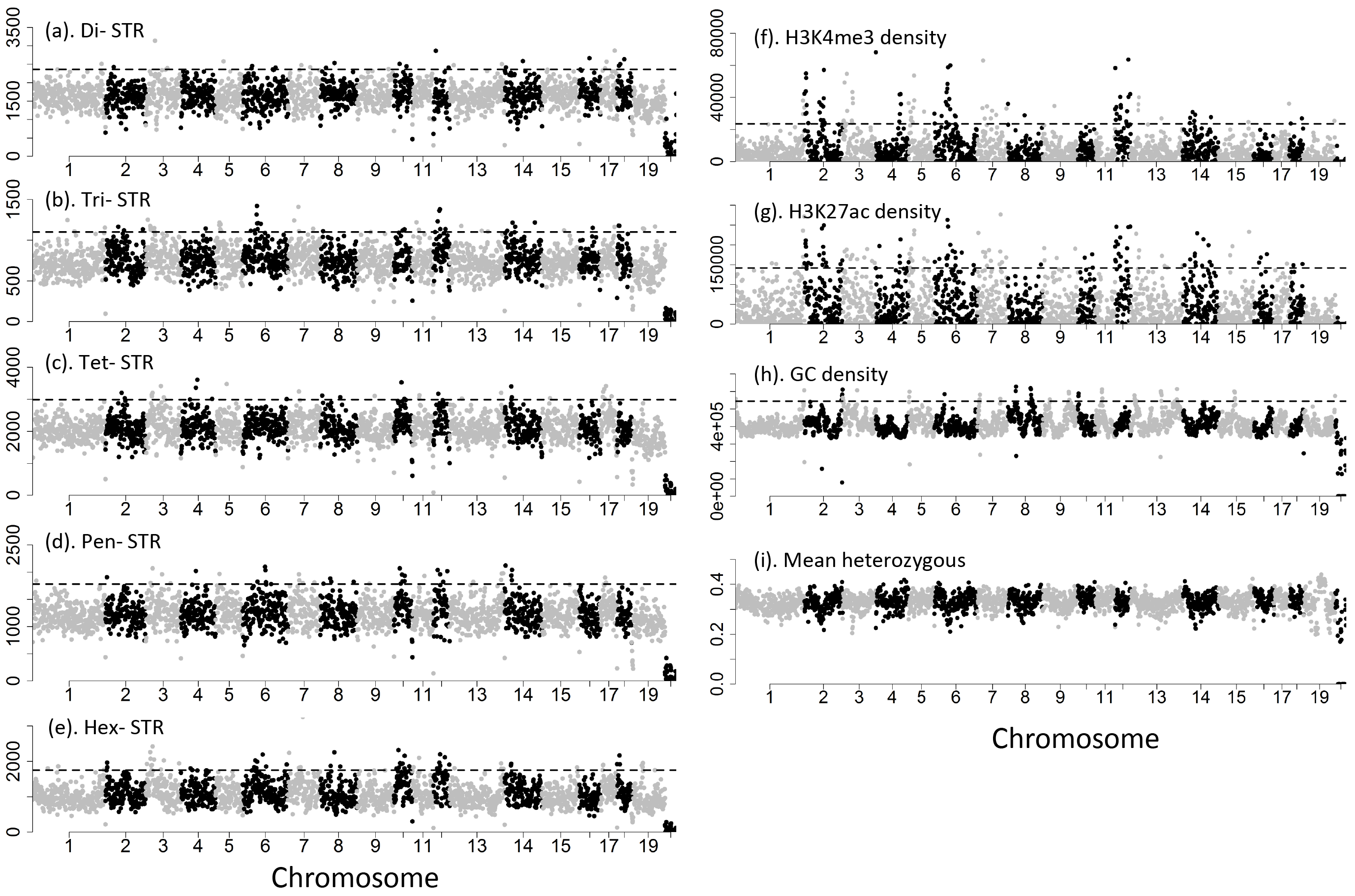

Supplement: Supplementary file 8 — Additional file 8: Figure S5. Distribution of different types of polymorphic short tandem repeats (STRs) by their unit lengths across the genome. The Manhattan plots show the distributions of the number of (a) Di-, (b) Tri-, (c) Tet, (d) Pen, (e) Hex STRs within 1-Mb sliding windows across the genome, the distribution of H3K4me3 (bp/Mb) (f), H3K27ac (bp/Mb) (g), GC content (bp/Mb) (h) and mean heterozygosity of the pSTRs in a 1 Mb sliding widows (i) were also included for comparison. [file 12711_2021_631_MOESM8_ESM.png]

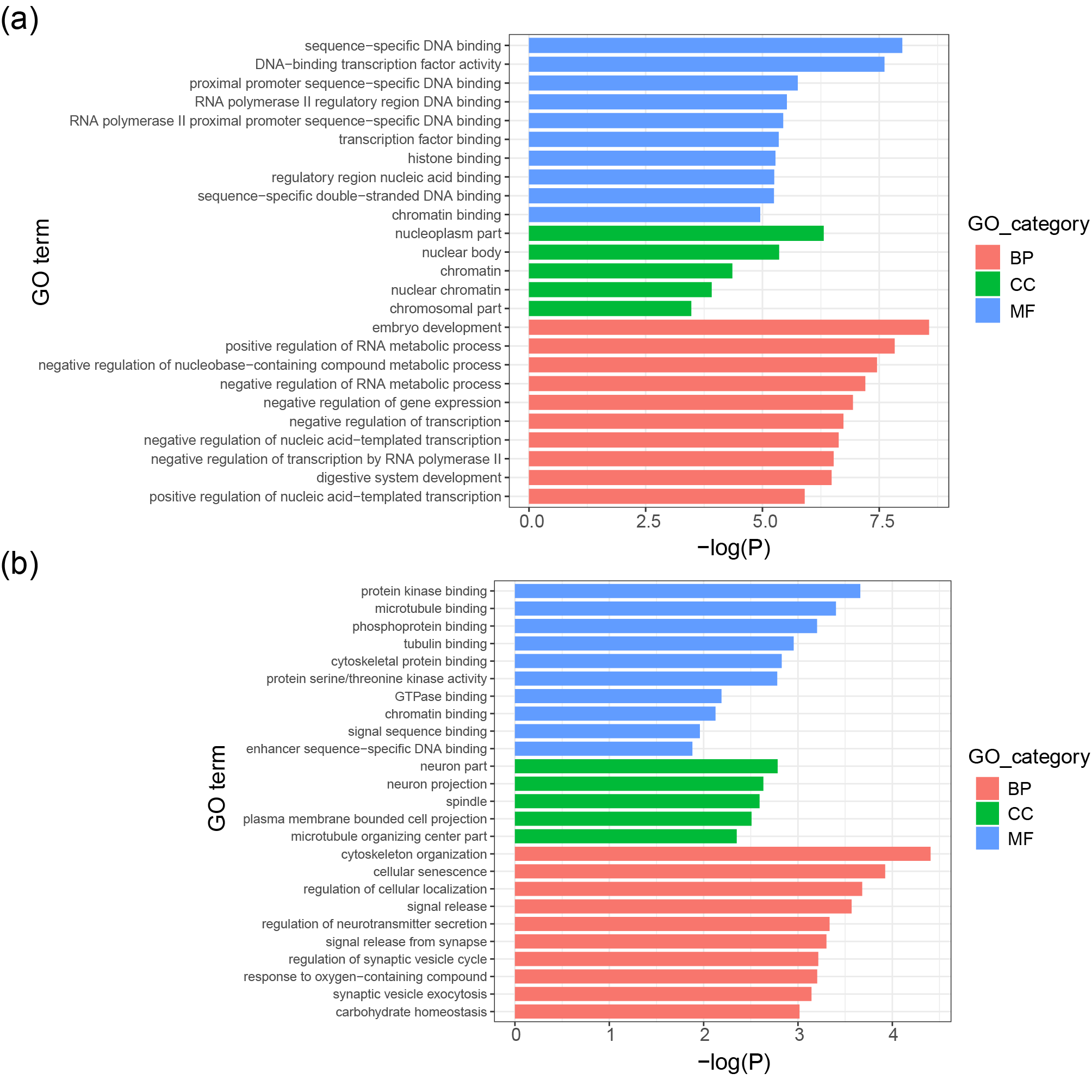

Supplement: Supplementary file 10 — Additional file 10: Figure S6. Gene ontology enrichment analysis on genes harboring trinucleotide polymorphic short tandem repeats (pSTRs) in their (a) CDS and (b) 5′UTR regions. BP stands for biological process, CC for cellular component and MF for molecular function. [file 12711_2021_631_MOESM10_ESM.png]

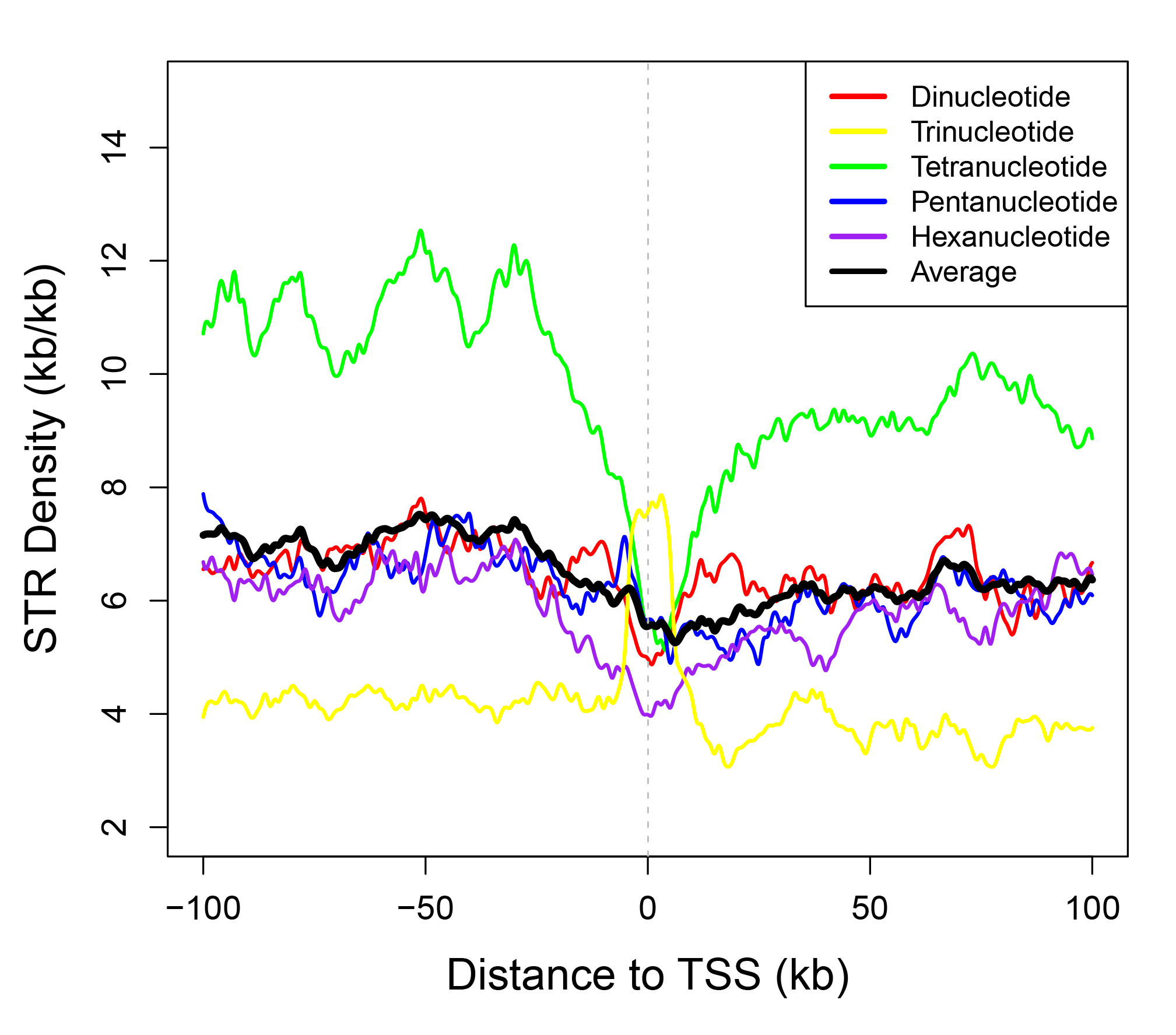

Supplement: Supplementary file 11 — Additional file 11: Figure S7. Density of different types of polymorphic short tandem repeats (pSTRs) in ± 100 bp flanking region of transcriptional start sites (TSS) across the genome. Different colors denote different types of pSTRs with unit lengths from 2 to 6. [file 12711_2021_631_MOESM11_ESM.png]

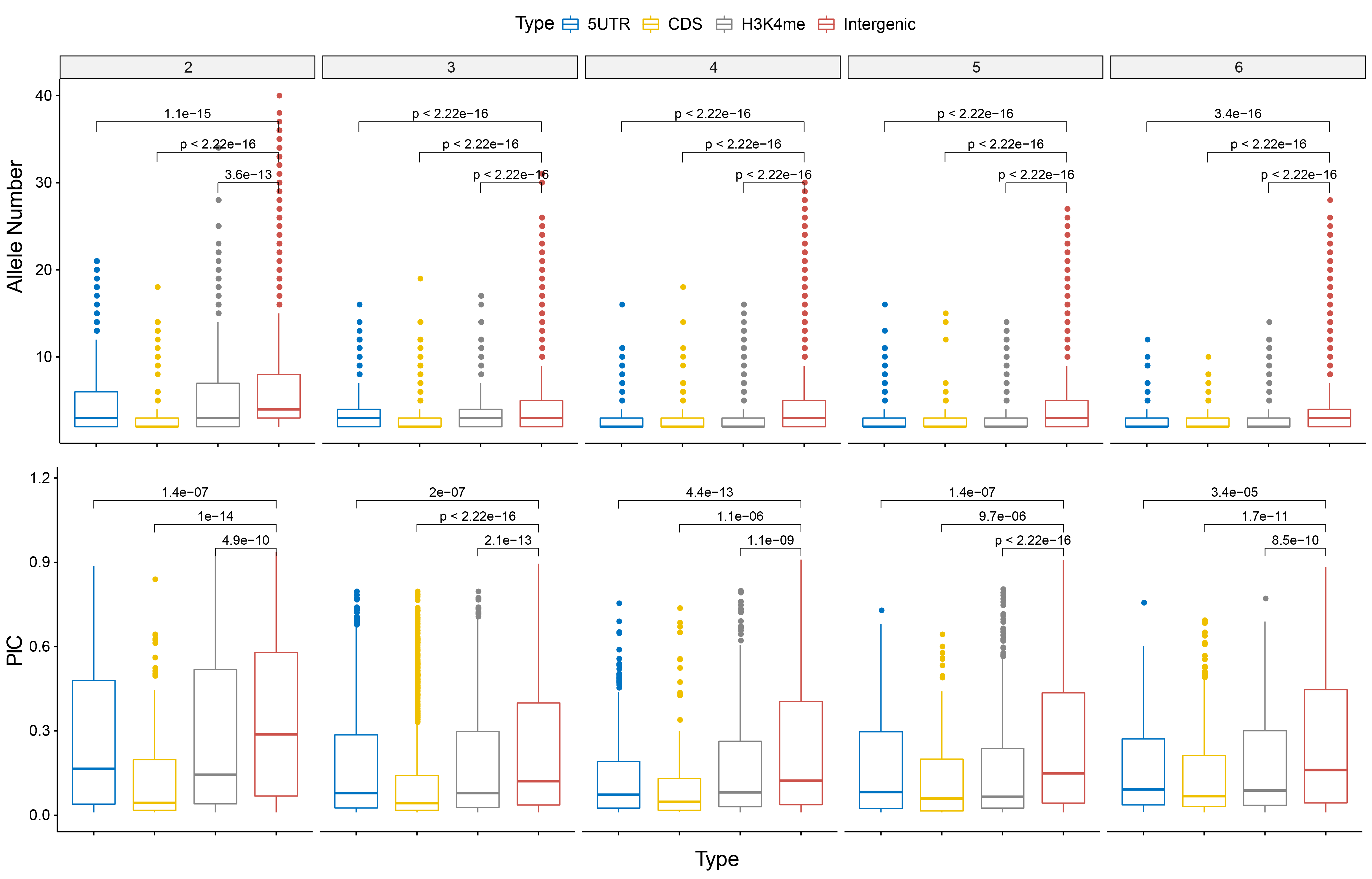

Supplement: Supplementary file 12 — Additional file 12: Figure S8. Genetic diversities (measured by allele number and polymorphic information content) of different types of polymorphic short tandem repeats with unit lengths from 2 to 6 in 5′UTR, CDS, H3K4me3 and intergenic regions. The p-value was estimated using wilcox.test in ggpubr package in R program. [file 12711_2021_631_MOESM12_ESM.png]

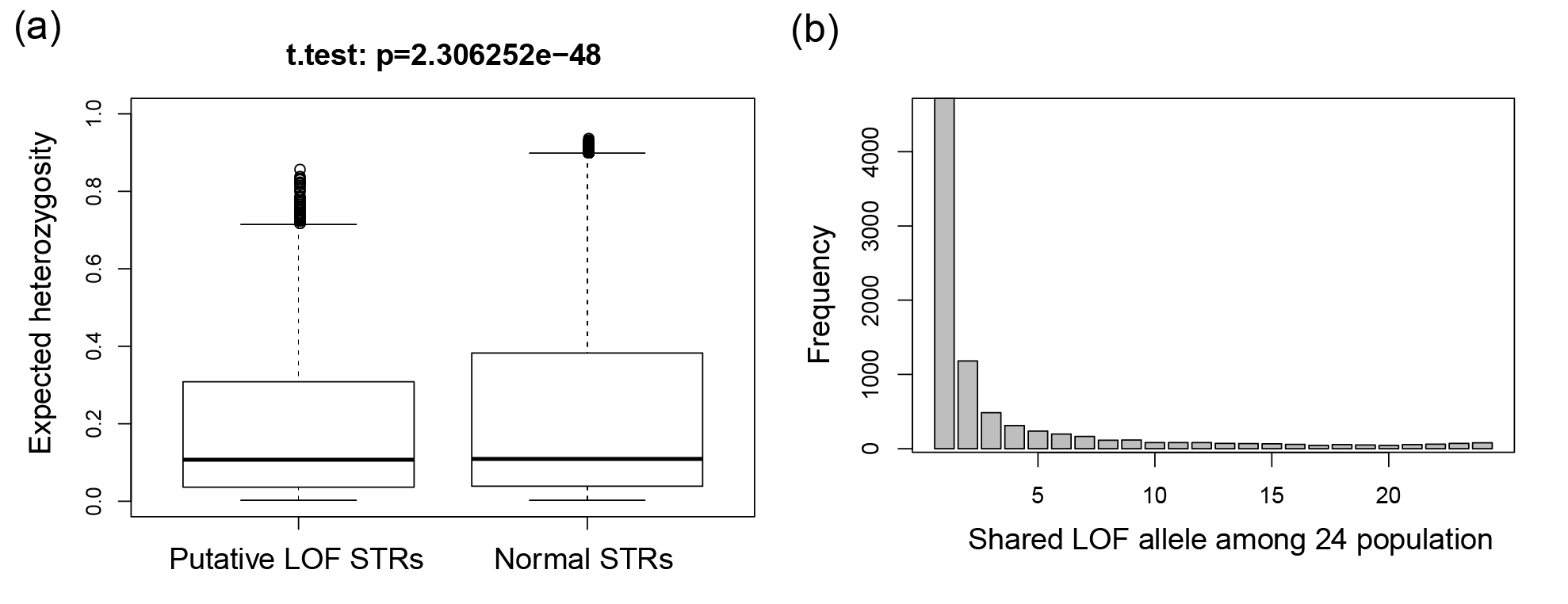

Supplement: Supplementary file 14 — Additional file 14: Figure S9. Properties of loss-of-function (LOF) polymorphic short tandem repeats (pSTRs) alleles. (a) Comparison of expected heterozygosity between LOF pSTRs and the other pSTRs. (b) Frequency distribution of LOF pSTRs alleles shared between populations. [file 12711_2021_631_MOESM14_ESM.png]

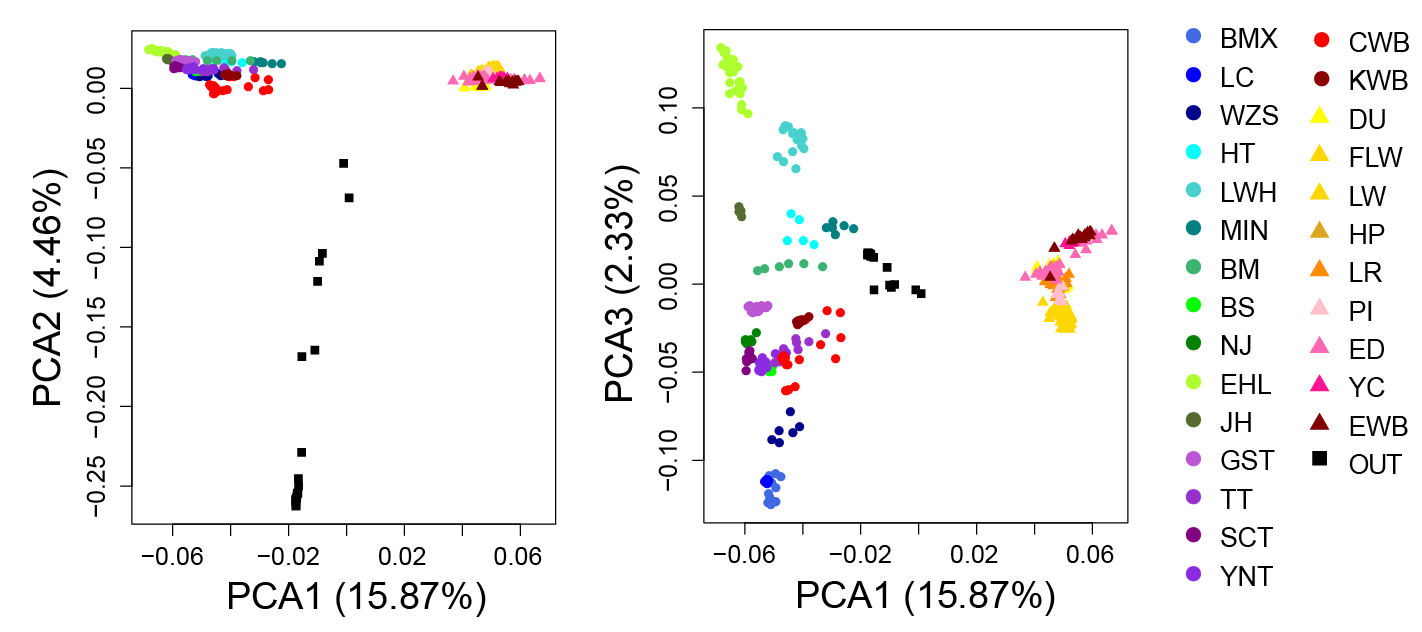

Supplement: Supplementary file 15 — Additional file 15: Figure S10. Principal component analysis (PCA) based on genome-wide STRs using 394 samples. The full names and abbreviations of the breeds/populations are as follow: Bamaxiang (BMX), Luchuan (LC), Wuzhishan (WZS), Laiwu (LWH), Hetao (HT), Min (MIN), Bamei (BM), Baoshan (BS), Neijiang (NJ), Jinhua (JH), Erhualian (EHL), Yunnan Tibetan (YT), Sichuan Tibetan (ST), Gansu Tibetan (GT), Tibet Tibetan (TT), Asian wild boars (AWB), European wild boars (EWB), European domestic pigs (ED), Duroc (DU), Landrace (LR), Pietrain (PI), Hampshire (HP), Large White (LW), French Large White (FLW), Yucatan (YC) and Outgroups (OUT). Among them, BMX, LC, WZZ, LWH, HT, MIN, BM, BS, NJ, JH, EHL, YT, ST, GT and TT are Asian domestic pigs. DU, LR, PI HP, LW, FLW, YC are European commercial pigs. [file 12711_2021_631_MOESM15_ESM.png]

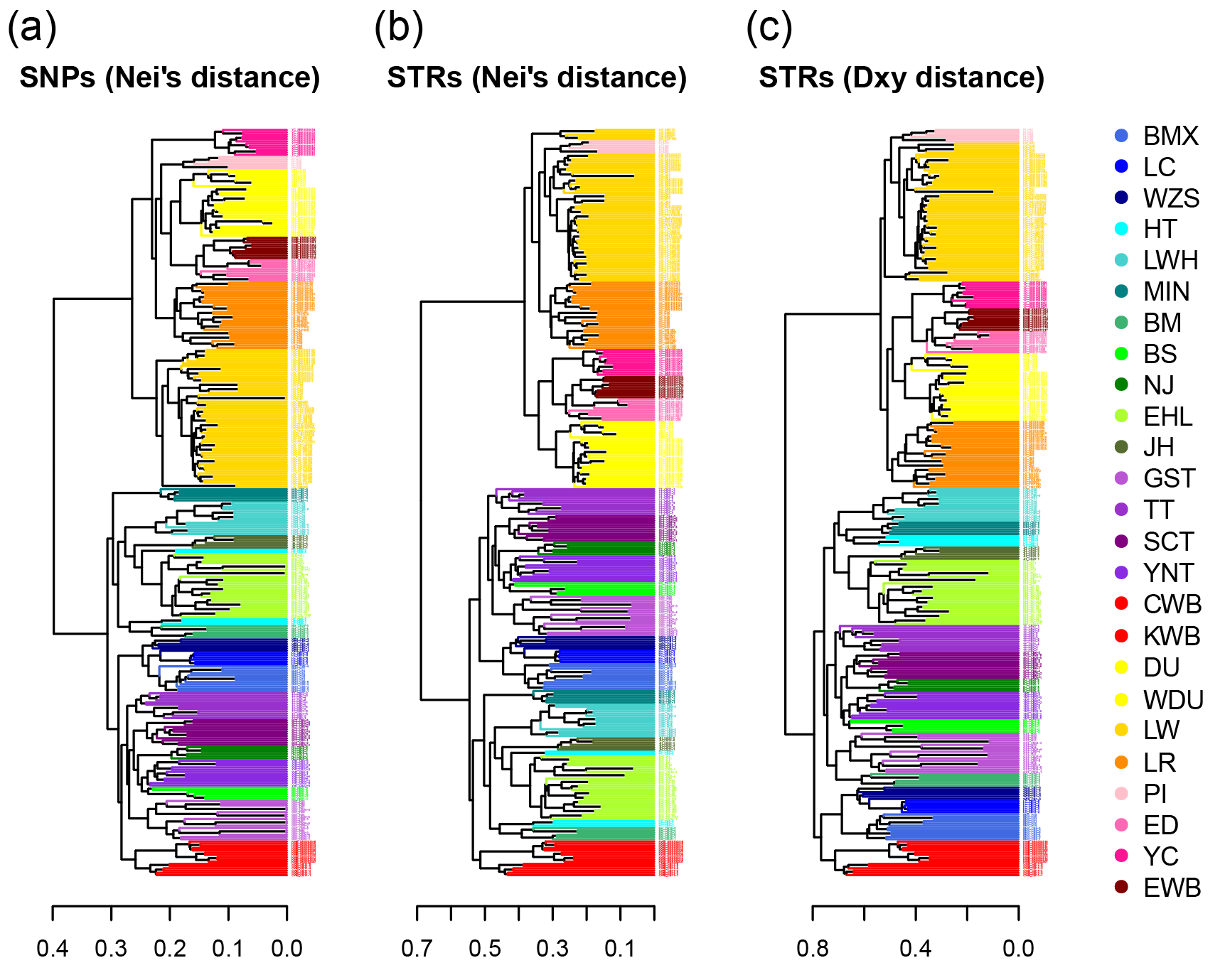

Supplement: Supplementary file 16 — Additional file 16: Figure S11. Genome-wide SNP and STRs clustering of multiple breeds using unsupervised clustering (method = complete). The colors show the breeds/populations to which the sample belongs. Only breeds with more than five samples were kept for analysis. The full names and abbreviations of the breeds/populations are as follow: Bamaxiang (BMX), Luchuan (LC), Wuzhishan (WZS), Laiwu (LWH), Hetao (HT), Min (MIN), Bamei (BM), Baoshan (BS), Neijiang (NJ), Jinhua (JH), Erhualian (EHL), Yunnan Tibetan (YT), Sichuan Tibetan (ST), Gansu Tibetan (GT), Tibet Tibetan (TT), Asian wild boars (AWB), European wild boars (EWB), European domestic pigs (ED), Duroc (DU), Landrace (LR), Pietrain (PI), Hampshire (HP), Large White (LW), French Large White (FLW), Yucatan (YC) and Outgroups (OUT). Among them, BMX, LC, WZZ, LWH, HT, MIN, BM, BS, NJ, JH, EHL, YT, ST, GT and TT are Asian domestic pigs. DU, LR, PI HP, LW, FLW, YC are European commercial pigs. [file 12711_2021_631_MOESM16_ESM.png]

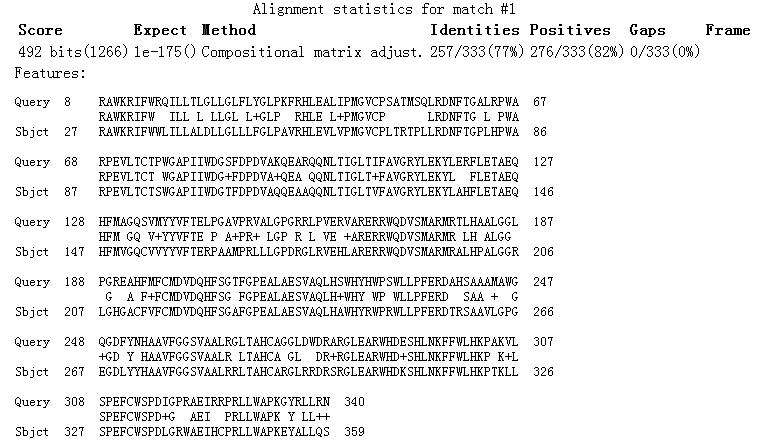

Supplement: Supplementary file 17 — Additional file 17: Figure S12. Comparison of the human protein sequence (Query sequence, NP_001073907) with the porcine protein sequence (Subject Sequence, XP_020951514.1) using BLASTP. [file 12711_2021_631_MOESM17_ESM.jpg]

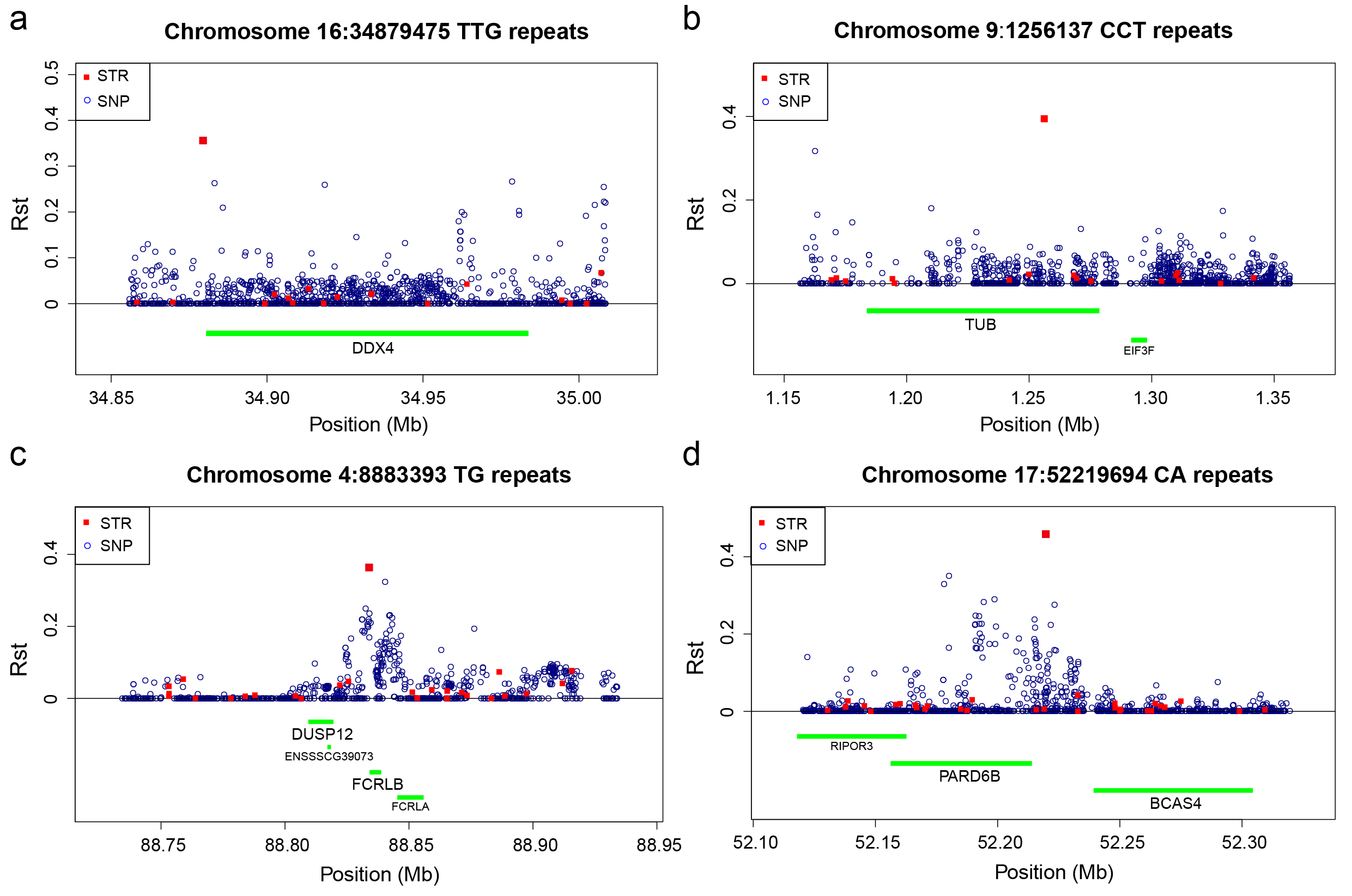

Supplement: Supplementary file 18 — Additional file 18: Figure S13. Regional signatures of population differentiation between domestic pig and wild boars in Asia (a) and Europe (b–d), where the STRs showed higher ${\text{R}}_{{{\text{st}}}}$Rst values than SNPs within ± 100 kb flanking regions. [file 12711_2021_631_MOESM18_ESM.png]

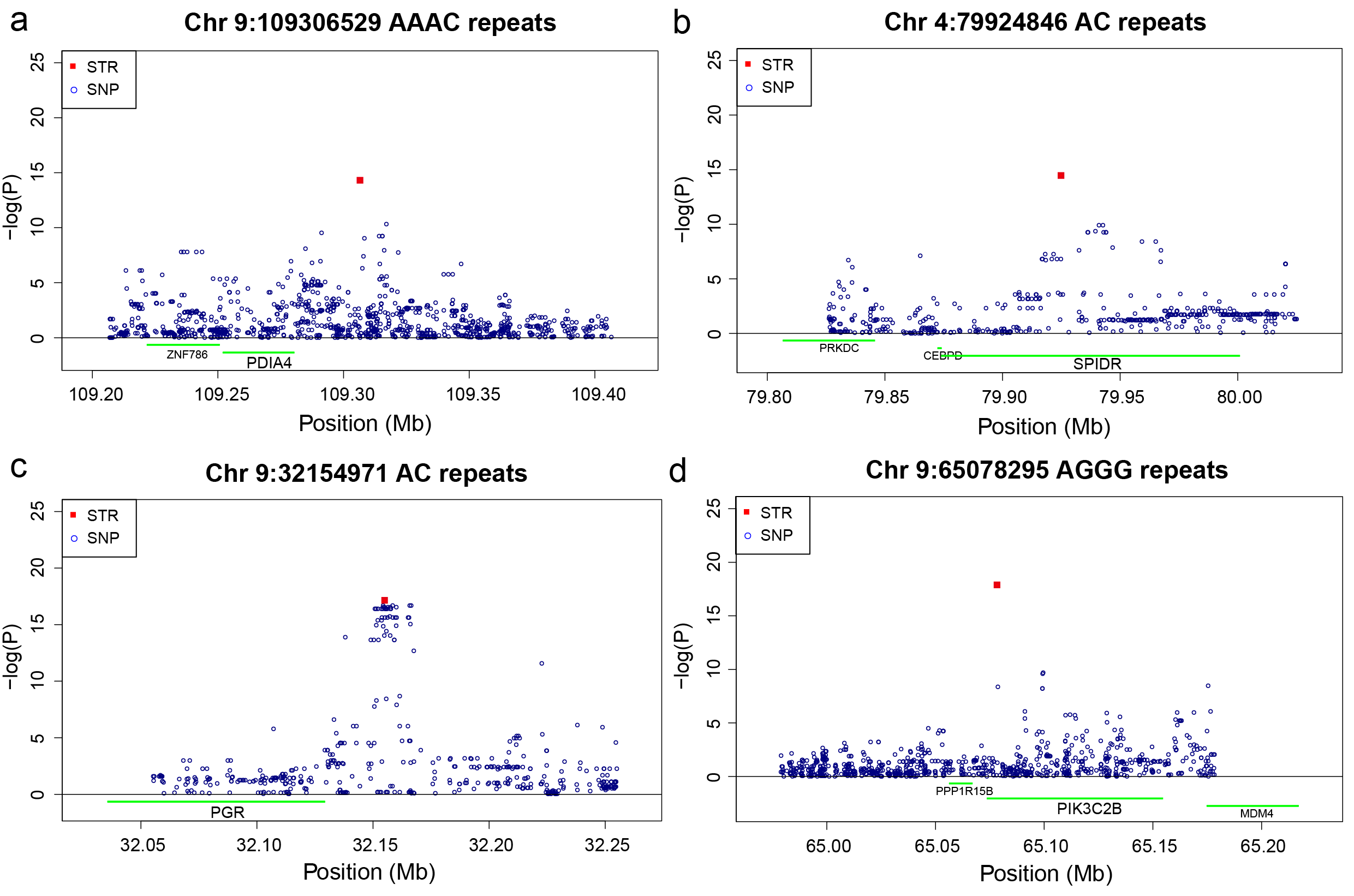

Supplement: Supplementary file 25 — Additional file 25: Figure S14. Regional plots of association signals of pSTRs with annual mean temperature (a, b) and altitude (c, d) among Chinese indigenous pig populations, for which STRs show stronger associations than SNPs within ± 100 kb flanking regions. [file 12711_2021_631_MOESM25_ESM.png]
